# Carnosine quenches the reactive carbonyl acrolein in the central nervous system and attenuates autoimmune neuroinflammation

**DOI:** 10.1186/s12974-021-02306-9

**Published:** 2021-11-05

**Authors:** Jan Spaas, Wouter M. A. Franssen, Charly Keytsman, Laura Blancquaert, Tim Vanmierlo, Jeroen Bogie, Bieke Broux, Niels Hellings, Jack van Horssen, Dheeraj Kumar Posa, David Hoetker, Shahid P. Baba, Wim Derave, Bert O. Eijnde

**Affiliations:** 1 University MS Center (UMSC) Hasselt – Pelt, Hasselt, Belgium; 2 BIOMED Biomedical Research Institute, Faculty of Medicine and Life Sciences, Hasselt University, Hasselt, Belgium; 3 Department of Movement and Sports Sciences, Faculty of Medicine and Health Sciences, Ghent University, Ghent, Belgium; 4 REVAL Rehabilitation Research Center, Faculty of Rehabilitation Sciences, Hasselt University, Hasselt, Belgium; 5 Neuro-Immune Connections and Repair Lab, Department of Immunology and Infection, Biomedical Research Institute, Hasselt University, Diepenbeek, Belgium; 6 Division of Translational Neuroscience, Department Psychiatry and Neuropsychology, European Graduate School of Neuroscience, School for Mental Health and Neuroscience, Maastricht University, Maastricht, The Netherlands; 7 Department of Internal Medicine, Cardiovascular Research Institute Maastricht, Maastricht University, Maastricht, The Netherlands; 8 Department of Molecular Cell Biology and Immunology, Amsterdam Neuroscience, MS Center Amsterdam, Amsterdam University Medical Center, Location VUmc, Amsterdam, The Netherlands; 9 Diabetes and Obesity Center, University of Louisville, Louisville, KY USA

**Keywords:** Acrolein, Carnosine, Multiple sclerosis, Neuroinflammation, Oxidative stress, Reactive carbonyl

## Abstract

**Background:**

Multiple sclerosis (MS) is a chronic autoimmune disease driven by sustained inflammation in the central nervous system. One of the pathological hallmarks of MS is extensive free radical production. However, the subsequent generation, potential pathological role, and detoxification of different lipid peroxidation-derived reactive carbonyl species during neuroinflammation are unclear, as are the therapeutic benefits of carbonyl quenchers. Here, we investigated the reactive carbonyl acrolein and (the therapeutic effect of) acrolein quenching by carnosine during neuroinflammation.

**Methods:**

The abundance and localization of acrolein was investigated in inflammatory lesions of MS patients and experimental autoimmune encephalomyelitis (EAE) mice. In addition, we analysed carnosine levels and acrolein quenching by endogenous and exogenous carnosine in EAE. Finally, the therapeutic effect of exogenous carnosine was assessed in vivo (EAE) and in vitro (primary mouse microglia, macrophages, astrocytes).

**Results:**

Acrolein was substantially increased in inflammatory lesions of MS patients and EAE mice. Levels of the dipeptide carnosine (β-alanyl-l-histidine), an endogenous carbonyl quencher particularly reactive towards acrolein, and the carnosine-acrolein adduct (carnosine-propanal) were ~ twofold lower within EAE spinal cord tissue. Oral carnosine treatment augmented spinal cord carnosine levels (up to > tenfold), increased carnosine-acrolein quenching, reduced acrolein-protein adduct formation, suppressed inflammatory activity, and alleviated clinical disease severity in EAE. In vivo and in vitro studies indicate that pro-inflammatory microglia/macrophages generate acrolein, which can be efficiently quenched by increasing carnosine availability, resulting in suppressed inflammatory activity. Other properties of carnosine (antioxidant, nitric oxide scavenging) may also contribute to the therapeutic effects.

**Conclusions:**

Our results identify carbonyl (particularly acrolein) quenching by carnosine as a therapeutic strategy to counter inflammation and macromolecular damage in MS.

**Supplementary Information:**

The online version contains supplementary material available at 10.1186/s12974-021-02306-9.

## Background

In multiple sclerosis (MS), the infiltration of circulating lymphocytes and monocytes triggers sustained central nervous system (CNS) inflammation leading to demyelination and axonal damage [1–3]. Mounting evidence supports that reactive oxygen species (ROS) mediate CNS injury in both the acute and chronic disease stage [4, 5]. The abundance of lipids renders the CNS extremely vulnerable to lipid peroxidation, a chain reaction of repetitive hydrogen abstractions from poly-unsaturated fatty acids (PUFAs) resulting in the formation of reactive carbonyl species. Lipid-derived reactive carbonyls, including 4-hydroxy-2-nonenal (HNE) and acrolein, have a relatively long half-life and exert strong cytotoxic effects by covalently binding with surrounding nucleophilic macromolecules, i.e. proteins, RNA, DNA and lipids [6, 7]. In fact, it has become increasingly clear that reactive carbonyl species are not merely surrogate markers of oxidative injury but can mediate and aggravate disease progression by themselves. Accordingly, therapeutic strategies to prevent carbonyl (over)load are emerging for numerous disorders, such as diabetes/obesity [8, 9]. Even though elevated levels of lipid-derived carbonyls have already been reported in MS [10–15], very little is known about the processes underlying their generation, potential pathological role, and detoxification, nor on the therapeutic benefits of carbonyl quenchers to counter CNS inflammation and macromolecular damage.

A variety of small molecules with the ability to quench and eliminate reactive carbonyl species are endogenously present in cells (phase II deactivation). The reactivity of different quenchers is dependent on the type of reactive carbonyl involved [16]. Carnosine (β-alanyl-l-histidine) is a naturally occurring histidine-containing dipeptide (HCD) with carbonyl quenching properties linked to its amino group and imidazole ring [17, 18]. Alongside one or more related HCDs, such as homocarnosine, anserine or balenine, carnosine is predominantly found in excitable tissues (CNS and muscle), where it is synthetized by the enzyme carnosine synthase (CARNS1) [17, 19]. Carnosine is especially reactive towards α,β-unsaturated carbonyls [6, 20–22], of which acrolein is the strongest electrophile (approximately 100-fold more than HNE) [7, 23]. Molecular modelling studies and advances in detection techniques have yielded important insights in the formation and metabolism of carnosine-carbonyl adducts in vitro and in vivo [8, 16, 24–26]. Conjugation of carnosine to acrolein generates the oxidized product carnosine-propanal, which can be further reduced to carnosine-propanol before renal excretion [20]. By acting as a sacrificial nucleophile, exogenous carnosine administration has been suggested to slow the progression of oxidative-driven diseases [17, 18]. The ability to cross the blood–brain barrier [27] and the favorable toxicological profile [28, 29] of this naturally occurring molecule underscore its therapeutic potential for CNS diseases.

Here, we studied the generation of acrolein during neuroinflammation and its quenching by endogenous and exogenous carnosine. Our data demonstrate significant acrolein-protein adduct formation in inflammatory lesions of MS patients and experimental autoimmune encephalomyelitis (EAE) mice, a model for autoimmune neuroinflammation that mimics MS. In EAE, this was associated with depletion of carnosine in spinal cord tissue, whereas oral carnosine intake augmented acrolein quenching and reduced inflammation and macromolecular damage. Our results identify carbonyl (in particular acrolein) quenching by carnosine as a therapeutic strategy for neuroinflammatory disorders such as MS.

## Materials and methods

### Acrolein-protein adducts in active MS lesions

Frozen brain material from active MS lesions was obtained from The Netherlands Brain Bank. Clinical patient details are depicted in Additional file 1: Table S1. Following acetone fixation (10 min) and blocking (30 min), serial cryosections were stained overnight for proteolipid protein (PLP) to assess demyelination and CD68 or HLA-DR to assess the presence and distribution of microglia/macrophages [30]. Staining was visualized with HRP-labelled secondary antibodies (Envision+, Dako) and 3,3′-Diaminobenzidine (DAB) substrate-chromogen (Dako). Similarly, sections were stained with antibodies against protein-bound acrolein (1/100, mouse monoclonal, 10A10, Novus Biologicals). For fluorescent double-staining, acrolein-protein adducts were visualized with secondary goat anti-mouse (1/600, AF 555, A-21425, Thermo Fisher), followed by overnight incubation with primary antibodies against CD68 (1/100, mouse monoclonal, M0814 KP1 clone, Dako) or glial fibrillary acidic protein (GFAP, 1/100, mouse monoclonal, G3893, Sigma), and secondary goat anti-mouse (1/400, IgG1 AF 488, A-21121, Thermo Fisher). Imaging was performed with a Leica DM4000 B LED (Leica Microsystems).

### Animals and EAE induction

Female 9-to-11 week old C57BL/6 OlaHSD mice (Envigo) were actively immunized with two subcutaneous injections of 100 µL myelin oligodendrocyte glycoprotein peptide fragment 35–55 emulsified in complete Freund’s adjuvant (MOG_35–55_/CFA) in the upper and lower back. Intraperitoneal injections of 100 µL pertussis toxin in phosphate-buffered saline (PBS) were administered immediately hereafter and 24 h later (EK-2110, Hooke Laboratories, MA). Clinical symptoms (scale 0–5, including 0.5 increments: 0, no symptoms; 1, limp tail; 2, hindlimb paresis; 3, hindlimb paralysis; 4, front limb paresis; 5, death due to EAE) and body weight (g) were monitored daily by a blinded assessor. Mice were housed on a 12 h:12 h light:dark cycle under standard room conditions (temperature 20–24 °C, relative humidity 30–60%) with ad libitum access to drinking water and food pellets (Teklad 2018C, Envigo). In a separate experiment, carnosine treatment was investigated in a monophasic EAE model using female Lewis rats, as described previously (see Ref. [31] and Additional file 2: Table S2).

### EAE study procedures

Healthy control and EAE mice were randomly allocated to different intervention groups. l-Carnosine treatment was administered by dissolving 3 g/L (0.3%), 15 g/L (1.5%) or 30 g/L (3%) carnosine in the drinking water, starting 7 days prior to EAE induction until the end of the study period. Water bottles were refreshed every 2–3 days. l-Carnosine was a gift from Flamma (Flamma Group, Italy). EAE mice were sacrificed at the disease peak (acute EAE, days 14–18 post immunization), following partial recovery (subacute EAE, days 24–28) or in the chronic stage (day 56). Control mice were sacrificed along with the acute EAE mice. Following overdose injection of Dolethal (200 mg/kg, i.p.), blood was collected from the right ventricle with heparin-coated syringes. Mice were perfused with PBS/heparin (25 UI/mL) via a left ventricular puncture and spinal cords were isolated. Blood samples were centrifuged (5 min, 3500 rpm) and plasma was stored at − 80 °C. Spinal cords were cut in four segments that were placed immediately in liquid nitrogen or first embedded in Tissue-Tek O.C.T. compound, and stored at − 80 °C. A graphical summary of the experimental designs used for the different analyses presented in this manuscript is shown in Additional file 4: Fig. S1.

### Detection of HCDs and carnosine-carbonyl adducts by UPLC-ESI–MS/MS

Ultrahigh-performance liquid chromatography electrospray ionization tandem mass spectrometry (UPLC-ESI–MS/MS) was used to detect HCDs and their carbonyl conjugates. A 5% homogenate was prepared from the thoracic spinal cord (18.5 ± 3.8 mg wet tissue weight (WW)) in extraction solution containing HCl (10 mM) and internal standards tyrosyl-histidine (10 µM, Bachem), l-carnosine-d4 (10 µM, CDN Isotopes) and l-anserine-d4 (10 µM, CDN Isotopes). Following homogenisation (30 s, 3 m/s, Bead Ruptor Elite, Omni International), samples were sonicated on ice, centrifuged at 4 °C (10 min, 16,000×*g*), and supernatants were stored at − 80 °C. Standards containing carnosine (1 mM, Sigma), anserine (1 mM, Sigma) and homocarnosine (1 mM, synthesized by Mike Wempe, PhD, University of Colorado) were prepared in extraction solution and were serially diluted (1:1) 19 times. Before analysis, standards and samples (spinal cord or plasma) were diluted 50 × in a 75:25 acetonitrile:water mixture and vortexed. UPLC-ESI–MS/MS was performed by injecting 5 µL sample into a Waters ACQUITY UPLC H-Class System coupled with a Xevo TQ-S micro triple quadrupole. The analytes were first separated by a Waters Acquity BEH HILIC column (1.7 μm, 2.1 × 50 mm) equipped with an in-line frit filter unit and then analysed by mass spectrometry in the positive mode. The analytes were eluted by using a binary solvent system consisting of 10 mM ammonium formate, 0.125% formic acid in 5:95 acetonitrile:water (mobile phase A) or 95:5 acetonitrile:water (mobile phase B) at a flow rate of 0.55 mL/min. Initial conditions were 0.1:99.9 (A:B), ramping to 99.9:0.1 (A:B) over 5 min and then quickly ramping to 0.1:99.9 (A:B) over 0.5 min.

Chromatograms were acquired using the transitions: carnosine 227 → 110 m*/z*, homocarnosine 241 → 156 m*/z*, anserine 241 → 109 m*/z*, carnosine-propanal 283 → 166 m*/z*, carnosine-propanol 285 → 110 m*/z*, carnosine-HNE 383 → 110 m*/z*, homocarnosine-acrolein 297 → 110 m*/z*, glutathione (GSH) 308 → 179 m*/z*, tyrosyl-histidine 319 → 110 m*/z*, carnosine-d4 231 → 110 m*/z*, anserine-d4 245 → 110 m*/z* in multiple reaction monitoring (MRM) mode. The AUC of carnosine, homocarnosine and carnosine-propanal were expressed relative to AUC of internal standard l-carnosine-d4, anserine relative to l-anserine-d4 and GSH relative to tyrosyl-histidine. Analyte levels were calculated from the standard curve (carnosine, homocarnosine, anserine) or from the internal standard and expressed as nmol/mgWW. Sample protein content was determined to express analyte levels per mg protein (data available upon request). The lower limit of quantitation (LOQ) for carnosine was 24.4 nM, homocarnosine 48.9 nM, and anserine 24.4 nM. The coefficient of variation (CV) of HCD measurements in spinal cord was determined using replicates within one sample run (n = 6) and between different sample runs (n = 3). Based on the calculation of total within-laboratory precision outlined by Chesher et al*.* [32], the CV% of carnosine was 1.72%, homocarnosine 3.01% and anserine 4.70%.

### Acrolein- and HNE-protein adducts by western blot

In order to quantify acrolein-protein and HNE-protein adducts by western blot, lumbrosacral spinal cord segments were manually homogenized on ice in RIPA buffer (50 mM Tris pH 8.0, 150 mM NaCl, 0.5% sodium deoxycholate, 0.1% SDS, 1% Triton-X100) with freshly added protease and phosphatase inhibitors (Roche). Sample protein content was measured with Pierce BCA Protein Assay Kit (Thermo Fisher). Protein samples (35 µg) were diluted in loading buffer and separated in 10% polyacrylamide gels. Next, proteins were transferred to PVDF membranes (90 min, 350 mA) and blocked with 5% milk (acrolein) or 5% bovine serum albumin (HNE) for 1 h at room temperature. Membranes were stained overnight at 4 °C in the appropriate blocking buffer using antibodies against protein-bound acrolein (1/2000, mouse monoclonal, 10A10, Novus Biologicals) or -HNE (1/1000, rabbit polyclonal, ab46545, Abcam). Secondary HRP-conjugated antibodies were applied for 90 min (1/3000, room temperature), followed by chemiluminescent detection with Pierce ECL Plus Western Blotting Substrate (Thermo Fisher) in the Amersham Imager 680 (Cytiva). Between different steps, membranes were washed in TBS-T. Ponceau S (Sigma) was used to stain all membrane proteins. Protein bands (a.u.) were quantified with ImageJ software and normalized to Ponceau S intensity at the same molecular weight.

### Quantitative polymerase chain reaction (qPCR)

For gene expression analyses, total RNA was isolated from thoracic spinal cord segments using RNeasy Mini Kit (Qiagen). Briefly, samples were homogenized in 1 mL QIAzol reagent (Qiagen), mixed with chloroform and centrifuged (15 min, 12,000 rpm, 4 °C). The RNA layer was diluted in 70% ethanol and spinned down multiple times in the spin columns according to manufacturer’s instructions. Final RNA was diluted in RNAse-free water and concentration was measured with a NanoDrop spectrophotometer (Thermo Fisher) at 260 nm. Next, cDNA with a final concentration of 5 ng/µL was synthesized using 1000 ng RNA and qScript cDNA SuperMix (Quantabio). Real-time quantitative PCR (qPCR) was conducted on a StepOnePlus detection system (Applied Biosystems) using Fast SYBR Green Master Mix (Applied Biosystems). Primer sequences are detailed in Additional file 3: Table S3. The most stable reference genes were used for normalisation (geNorm) [33]. Analysis was performed with the 2^−ΔΔCt^ method and expressed as fold changes.

### Immunohistochemistry

Thoracolumbar spinal cord cryosections (10 µm) were used to stain T cells (CD3), microglia/macrophages (F4/80), astrocytes (GFAP), and acrolein-protein adducts. After acetone fixation (10 min) and blocking (30 min), sections were incubated with primary antibodies detecting F4/80 (1/100, overnight, rat monoclonal, MCA497G, Bio-Rad), CD3 (1/150, overnight, rat monoclonal, MCA500G, Bio-Rad), or GFAP (1/100, 3 h, mouse monoclonal, G3893, Sigma), followed by multiple washes and complementary secondary antibodies for 1 h (Thermo Fisher). Using fluorescent microscopy (Leica DM4000 B LED, Leica Microsystems), the entire spinal cord section was captured in a series of images. The total number of T cells or microglia/macrophages was calculated by dividing the entire positive stained area by the average area of one cell, and expressed per mm^2^ tissue (ImageJ) [34]. For astrocytes, the percentage GFAP^+^ area vs. total area was used (ImageJ). In a subset of experiments, acrolein-protein adducts (1/100, mouse monoclonal, 10A10, Novus Biologicals) were co-stained with F4/80 or GFAP. The acrolein-positive area (%) was analysed using ImageJ in entire spinal cord sections, and in lesion vs. non-lesion areas separately. Lesions were determined by hypercellularity (DAPI nuclear stain) and accumulation of F4/80^+^ cells. Negative control sections omitting one or all primary antibodies were included for all immunostainings.

### Cell culture

Primary microglial cell cultures were prepared from newborn (P0–2) C57BL/6 mouse pups. Briefly, brain cortices were freed from meninges, kept on ice, homogenised and dissociated to single-cell suspension with papain (20 U/mL, Sigma) and DNAse (0.15 mg/mL, Sigma). Mixed glial cell cultures were grown in PLL-coated flasks with DMEM D6429 (Sigma), 10% heat-inactivated fetal bovine serum (FBS, Biowest), 1% Penicillin–Streptomycin (PS, Sigma), at 37 °C, 5% CO_2_. On days 3, 7 and 11 after isolation, cell medium was fully replaced. Starting from day 3, L929 cell-conditioned medium (LCM) was added in a 1:2 ratio to promote microglial proliferation. On day 14, microglia were collected after shake-off procedure (230 rpm, 4 h), centrifuged (10 min, 300×*g*) and plated in DMEM containing 15% LCM. After shake-off, astrocytes were purified from mixed glial cultures over a period of 7–10 days by differential adhesion and mild trypsinization (Sigma). Mouse bone marrow-derived macrophages (BMDMs) were obtained from femoral and tibial bone marrow suspensions of 11-week-old C57BL/6 mice. BMDMs were differentiated for 7 days in RPMI 1640 medium (Lonza Bioscience) supplemented with 10% FBS, 0.5% PS, and 15% LCM (5% after plating) [35].

In all experiments, pre-treatment with l-carnosine (Flamma), l-anserine (Flamma), l-balenine (NNB Nutrition), β-alanine (Sigma), l-histidine (Sigma), tyrosyl-histidine (Bachem) or vehicle control (PBS) was given for 24 h (1–20 mM). Hereafter, cell medium was refreshed and cells were exposed to either lipopolysaccharide (LPS, 100 ng/mL, 24 h, Merck), hydrogen peroxide (H_2_O_2_, 100 µM, 24 h, VWR Chemicals), phorbol 12-myristate 13-acetate (PMA, 100 ng/mL, 15 min, Sigma), recombinant murine IFN-γ and IL-1β (100 ng/mL and 10 ng/mL, 24 h, PreproTech), or vehicle control (PBS). For determination of HCDs and their carbonyl conjugates (2e6 cells/flask), cells were washed, scraped, collected, centrifuged and stored at − 80 °C. Cell pellets were resuspended in extraction solution and processed for UPLC-ESI–MS/MS as described above. For gene expression analysis following LPS exposure (2e5 cells/well), cells were lysed in QIAzol (Qiagen), followed by RNA extraction, cDNA synthesis and qPCR amplification as described above. For flow cytometry (1.5e5 cells/well), LPS-stimulated cells were detached in PBS/EDTA (10 mM, 10 min), centrifuged, and stained with Fixable Viability Dye (FVD) eFluor 506 (1/1000, eBioscience) for 30 min at 4 °C. Next, cells were stained by F4/80-PeCy7 (1/100, Biolegend) and MHC class II-PE (1/500, eBioscience) for 15 min at room temperature. Flow cytometry was run on a BD LSRFortessa. Using FlowJo software (LLC, BD), the MHC-II mean fluorescence intensity was determined from the single cell, FVD^−^, F4/80^+^ population. To determine intracellular ROS levels (5e4 cells/well), PMA-stimulated cells were incubated with 2′,7′-Dichlorofluorescin diacetate (DCFH-DA, Sigma) for 30 min. Fluorescence spectroscopy detection with excitation/emission at 485/520 nm was performed immediately to measure the oxidized product 2′,7′-dichlorofluorescein (FLUOstar Optima, BMG Labtech). To determine nitrite release, an indicator of cellular nitric oxide production, culture medium from 1e5 cells/100 µL was obtained after LPS or IFN-γ/IL-1β exposure and 1:1 diluted with a mixture containing N-(1-Naphthyl)ethylenediamine dihydrochloride (Sigma) and Sulfanilamide (Sigma). The absorbance of the reaction product was read at 540 nm (iMark, Bio-Rad) and the concentration (µM) of nitrite was calculated from a linear standard curve containing Sodium Nitrite (Promega). All in vitro experiments were repeated ≥ 3 times.

### Statistics

Statistical analyses were performed with IBM SPSS Statistics v25 (NY, USA). Two independent groups were compared by t-tests or Mann–Whitney U tests, with Bonferroni-adjusted p values in case of multiple testing. Multiple groups were compared by one-way ANOVA and Tukey HSD post hoc tests, or Kruskal–Wallis with Bonferroni-adjusted pairwise comparisons. The time-dependent effect of carnosine treatment (Fig. 4) was analysed by two-way ANOVA (group × treatment, 4 × 2), followed by Bonferroni-corrected pairwise t-testing (per group) and one-way ANOVA (delta treatment values). AUC of clinical EAE scores was calculated in GraphPad Prism v7.04. Data are presented as mean ± SD, unless otherwise specified. *p < 0.05, **p < 0.01 for two-sided hypothesis testing.

## Results

### Acrolein-protein adduct formation in active MS lesions and EAE infiltrates

**Fig. 1:**
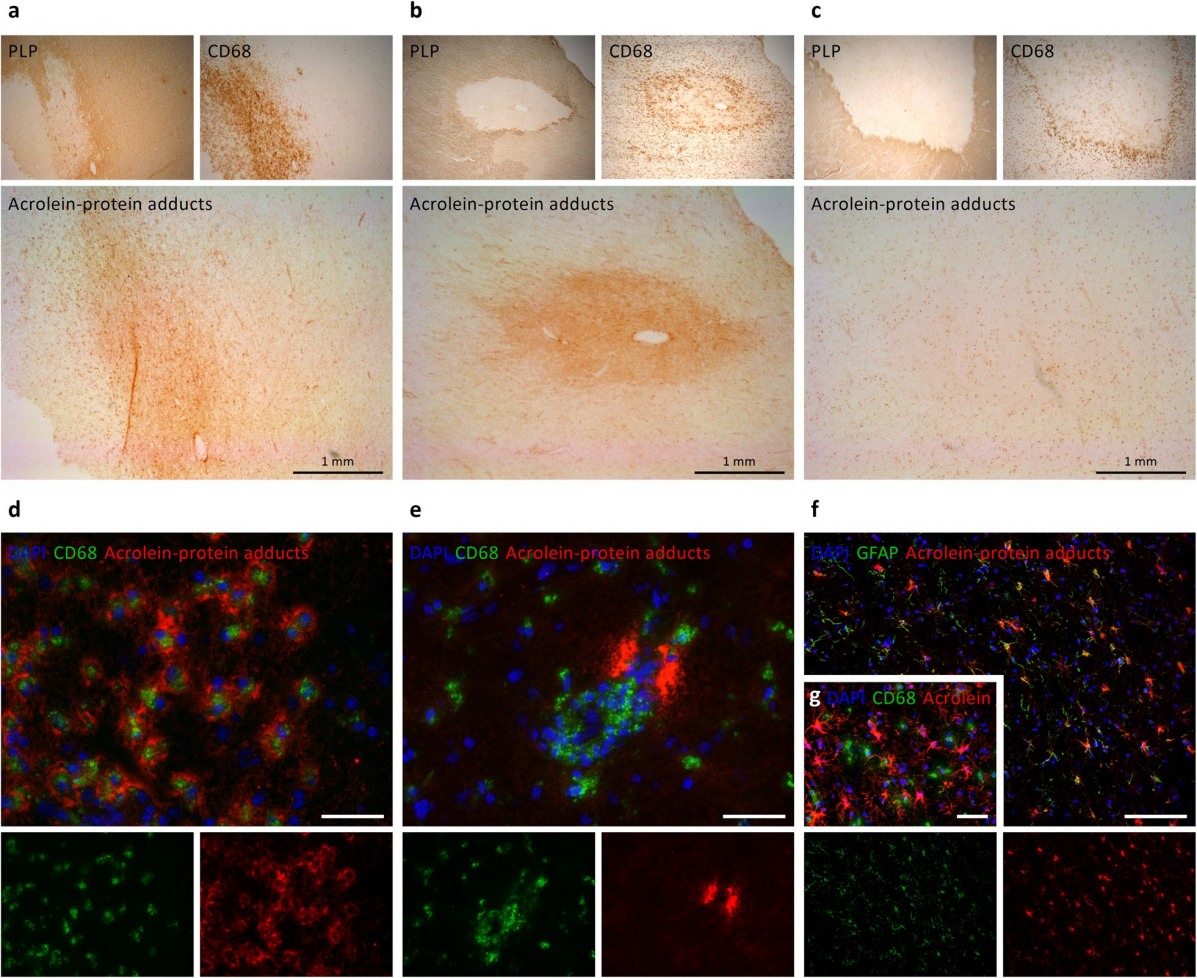
Acrolein-protein adduct formation in human MS lesions. Serial cryosections from demyelinated human MS lesions were stained against proteolipid protein (PLP), CD68 and acrolein-protein adducts. **a** Active MS lesion featuring CD68^+^ macrophages/microglia packed in the lesion center. Acrolein-protein adducts can be seen in the lesion. **b** Mixed active/inactive MS lesion containing CD68^+^ macrophages/microglia at the lesion border that enclose a hypocellular lesion center. Acrolein-protein adducts can be seen in the lesion. **c** Mixed active/inactive MS lesion with no evidence of acrolein-protein adduct formation. **d** Immunofluorescence image showing acrolein-protein adducts surrounding individual CD68^+^ macrophages/microglia in an MS lesion. **e** Acrolein-protein adducts in a perivascular cuff containing CD68^+^ macrophages/microglia. **f** Acrolein-protein adducts with a cellular morphology co-localized with GFAP immunoreactivity (astrocytes), but not with CD68 **(g)**. Scale bars are 1 mm (**a**–**c**), 50 µm (**d**–**g**) or 100 µm (**f**)

Excessive free radical production is a pathological hallmark of CNS inflammation in MS. To evaluate the downstream generation of acrolein, a highly reactive lipid-derived carbonyl that can induce significant protein modification [6], we examined acrolein-protein adduct formation in human brain samples containing demyelinated (PLP^−^) active MS lesions (with microglia/macrophages throughout the lesion area) and mixed active/inactive MS lesions (hypocellular lesion center with microglia/macrophages at the border) by immunohistochemistry [30]. Acrolein-protein adducts were present in both active and mixed active/inactive lesions (Fig. 1a, b), yet in some mixed active/inactive lesions no acrolein-protein immunoreactivity could be visualized via DAB staining (Fig. 1c). Notably, the pattern of acrolein staining was not uniform and varied between tissue blocks and between different areas of the same tissue block. In certain lesions, we observed acrolein-protein adducts surrounding densely packed CD68^+^ microglia/macrophages (Fig. 1d) or in perivascular cuffs (Fig. 1e). In addition, acrolein-protein adducts displaying a specific cellular morphology were repeatedly observed in the lesion center, lesion border, or even outside of the lesion (PLP^+^/CD68^−^ area). These cellular structures were identified as GFAP^+^ reactive astrocytes (Fig. 1f) and were CD68^−^ (Fig. 1g).

**Fig. 2:**
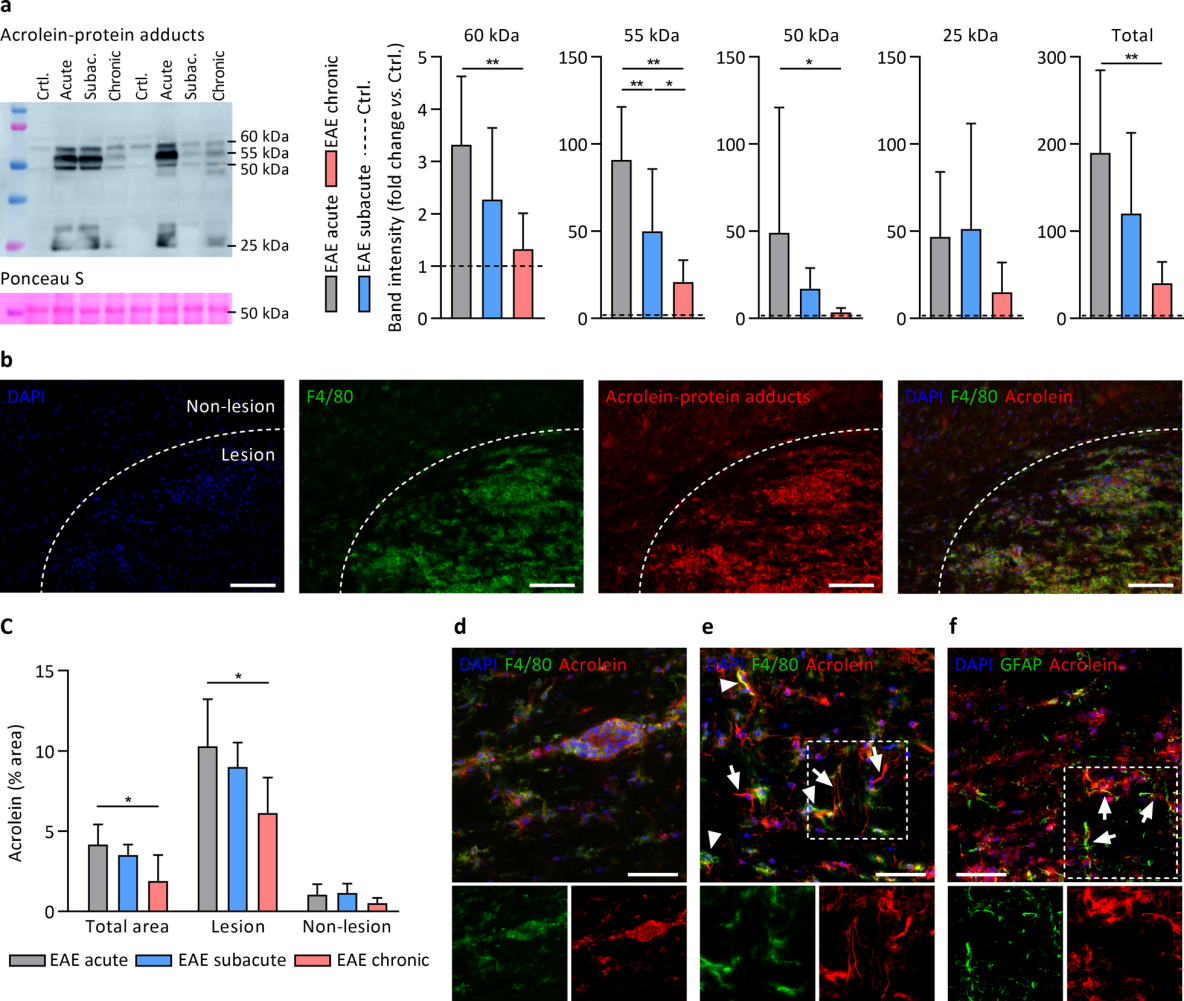
Acrolein-protein adduct formation coincides with high levels of inflammation and microglia/macrophage accumulation in different stages of EAE. **a** Detection of acrolein-protein adducts by western blot in different stages of EAE compared to healthy controls (dashed line). n = 7–8 animals/group. Acrolein-protein adducts were increased in EAE *vs.* healthy controls (statistics not shown), except for subacute and chronic EAE at 60 kDa, and chronic EAE at 50 kDa. **b** Immunohistochemistry demonstrating acrolein-protein adducts in hypercellular EAE spinal cord lesions characterized by an abundance of F4/80^+^ microglia/macrophages. **c** Quantification of acrolein-protein adduct immunoreactivity (% positive *vs.* total area) in EAE spinal cord sections, showing higher acrolein-protein levels in lesion *vs.* non-lesion areas, and in acute *vs.* chronic EAE. n = 6 animals/group. **d** Acrolein-protein adducts in perivascular cuff containing F4/80^+^ microglia/macrophages. **e** Acrolein-protein adducts in brain parenchyma co-localized with F4/80^+^ microglia/macrophages (arrowheads) and with astrocytic processes that appeared F4/80^−^ (arrows), but GFAP^+^ (arrows in **f**). Data are mean ± SD. One-way ANOVA or Kruskal–Wallis, post hoc testing *p < 0.05, **p < 0.01 between the indicated groups. Scale bars are 100 µm (**b**) or 50 µm (**d**–**f**)

We next examined whether acrolein- and HNE-protein adducts are increased within the spinal cord of EAE mice; an animal MS model featuring autoimmune neuroinflammation that is triggered by immunization with myelin antigens leading to lymphocyte and monocyte infiltration into the CNS [36]. Using a 0–5 clinical disability grading system (see methods), EAE mice displayed an initial disease peak (acute EAE, days 14–18 post immunization, clinical disability score 2.5 ± 1.1), followed by a slight remission (subacute EAE, days 24–28, score 1.9 ± 0.9) and stabilization (chronic EAE, day 56, score 2.2 ± 0.3). Spinal cord mRNA expression profiles (Additional file 4: Figure S2a) and accumulation of F4/80^+^ microglia/macrophages in spinal cord sections (Additional file 4: Fig. S2b) confirmed higher levels of inflammatory activity and oxidative/nitrosative stress in acute compared to chronic EAE. When examining spinal cord acrolein-protein adduct formation by western blot in EAE vs. control mice, increased acrolein-protein adducts were found at multiple molecular weights (25, 50, 55, 60 kDa) and at all EAE disease stages, with acute EAE mice exhibiting the greatest increase in acrolein damage (Fig. 2a). To further study the location of these adducts within the spinal cord, immunohistochemistry was performed and revealed that acrolein-protein adduct formation was predominantly present in inflammatory lesions in proximity to F4/80^+^ microglia and macrophages (Fig. 2b, c); both in the parenchyma and in perivascular cuffs (Fig. 2d, e). The fact that most acrolein immunoreactivity was found in or around such infiltrates suggests that the oxidative burst from inflammatory microglia/macrophages is a major source of acrolein accumulation in EAE spinal cord. Acrolein-protein adducts were also observed in structures with morphological features resembling astrocytic processes that were GFAP^+^ (Fig. 2f) but F4/80^−^ (Fig. 2e). We also examined whether the formation of HNE-protein adducts was increased in EAE spinal cords, but HNE-modified proteins were unaffected compared to control mice by western blot analysis (Additional file 4: Figure S3). Taken together, active MS lesions and inflammatory EAE infiltrates are featured by substantial levels of acrolein-protein adducts. These adducts were localized in and in close proximity to inflammatory microglia/macrophages, but also occurred in other structures such as astrocytes.

### Carnosine quenches acrolein in spinal cord but is depleted in EAE

**Fig. 3:**
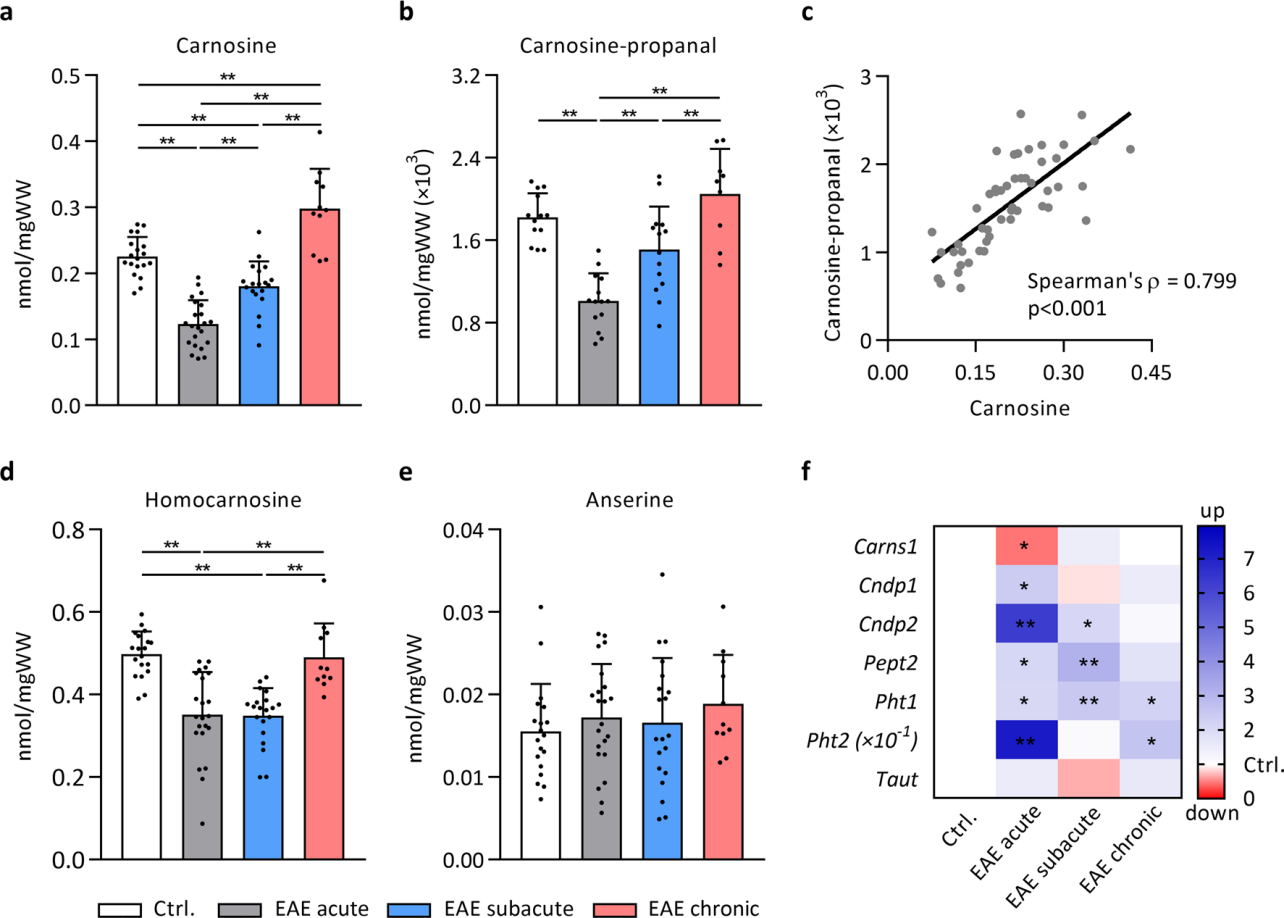
Spinal cord carnosine and carnosine-propanal are depleted in acute EAE. UPLC-ESI–MS/MS-based quantification of histidine-containing dipeptides and their carbonyl conjugates shows changes in **a, c** carnosine, **b, c** carnosine-propanal and **d** homocarnosine, but not **e** anserine, in different stages of EAE compared to healthy control mice. n = 8–23 animals/group. Carnosine-propanal values (~ 0.0015 nmol/mgWW) were transformed (× 10^3^) to facilitate visualisation on the Y axis. **f** mRNA expression of key regulators involved in carnosine homeostasis was altered in EAE spinal cord compared to healthy controls. Gene expression data are fold changes *vs.* control mice. Due to high expression of *Pht2* in EAE mice, data were transformed (× 10^–1^) to allow visualisation. n = 6–7 animals/group. *Carns1*, carnosine synthase; *Cndp1*, carnosine dipeptidase 1; *Cndp2*, cytosolic non-specific dipeptidase 2; *Pept2*, peptide transporter 2 (Slc15a2); *Pht1*, peptide/histidine transporter 1 (Slc15a4); *Pht2*, peptide/histidine transporter 2 (Slc15a3); *Taut*, taurine transporter (Slc6a6). Data are mean ± SD. One-way ANOVA or Kruskal–Wallis, post hoc testing *p < 0.05, **p < 0.01 between the indicated groups (**a**, **b**, **d**, **e**) or vs. healthy control mice (**f**)

Several endogenous quenchers and enzymes, which form conjugates and metabolize reactive carbonyls, are present in the CNS [20, 37, 38]. Here, we studied the histidine-containing dipeptides (HCDs), which exhibit high quenching activity towards acrolein [16, 22]. To determine whether the levels of these dipeptides and their carbonyl conjugates are affected within the spinal cord during different stages of EAE, an extensive profiling using UPLC-ESI–MS/MS was performed. The HCDs carnosine (0.23 ± 0.03 nmol/mgWW), homocarnosine (0.50 ± 0.06 nmol/mgWW) and anserine (0.016 ± 0.006 nmol/mgWW) were detected in mouse spinal cord, in addition to carnosine-propanal; the oxidized form of a carnosine-acrolein adduct (0.0018 ± 0.0002 nmol/mgWW). During acute EAE, carnosine (Fig. 3a) and carnosine-propanal (Fig. 3b) dropped to 0.12 ± 0.04 nmol/mgWW (− 46%) and 0.0010 ± 0.0003 nmol/mgWW (− 44%) respectively, before gradually recovering; indicating that carnosine homeostasis is challenged during EAE. Carnosine and carnosine-propanal levels strongly correlated with each other (Fig. 3c). Homocarnosine levels also decreased in acute and subacute EAE (− 30%, Fig. 3d), whereas anserine remained unchanged (Fig. 3e). No carnosine-propanol, carnosine-HNE or homocarnosine-acrolein conjugates were detected either in the healthy or EAE mice. Similar to carnosine, the levels of the endogenous antioxidant and carbonyl quencher GSH were decreased in (sub)acute EAE (− 30%, Additional file 4: Fig. S4).

HCD homeostasis is maintained by a complex interplay between synthesis, breakdown and transmembrane transport [17]. To gain insight in the mechanism by which the HCDs are depleted within the spinal cord of EAE mice, we next determined the spinal cord gene expression of key regulators involved in carnosine synthesis (*Carns1*), breakdown (*Cndp1*, *Cndp2*) and transport (*Pept2*, *Pht1*, *Pht2*, *Taut*). As shown in Fig. 3f, *Carns1* expression decreased (− 52%) in acute EAE, whilst the expression of carnosine-hydrolysing enzymes and transporters remained generally elevated throughout the disease course. Collectively, these data indicate that carnosine quenches acrolein to form carnosine-propanal in mouse spinal cord, and that endogenous carnosine homeostasis is disturbed during EAE, which is potentially linked to excessive acrolein load or alterations in the synthesis and breakdown of the dipeptide.

### Carnosine treatment increases carnosine and carnosine-propanal levels in a time-dependent manner in EAE spinal cord tissue

**Fig. 4:**
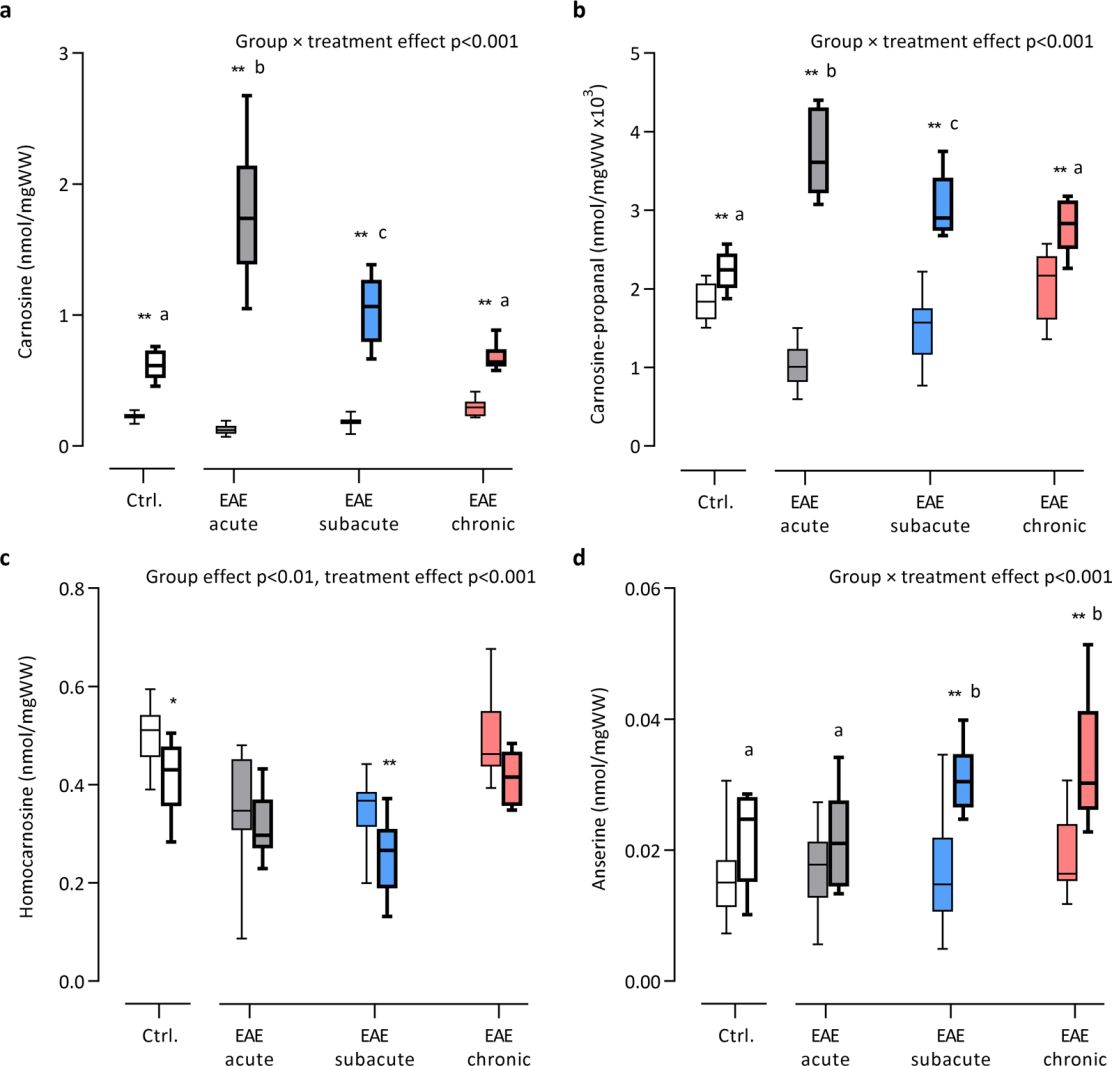
Time-dependent changes in spinal cord histidine-containing dipeptides following oral carnosine treatment in EAE. UPLC-ESI–MS/MS-based quantification of spinal cord **a** carnosine, **b** carnosine-propanal, **c** homocarnosine and **d** anserine following treatment with 1.5% carnosine (drinking water) in different stages of EAE and healthy controls. Box plots with bold lines represent carnosine-treated groups. n = 8–22 animals/group. Carnosine-propanal values (~ 0.0015 nmol/mgWW) were transformed (× 10^3^) to facilitate visualisation on the Y axis. Data are the median and minimum/maximum values. Results of two-way ANOVA (group × treatment, 4 × 2) are depicted on the figure. *p < 0.05, **p < 0.01 for comparison between the treated *vs.* respective untreated group (with Bonferroni correction). Characters above individual box plots (a/b/c) indicate which groups had a similar treatment response (delta values) in case of a significant interaction effect

Given that the spinal cord carnosine levels were decreased in EAE mice, we next studied if exogenous (oral) carnosine intake can increase the availability of carnosine in spinal cord tissue of mice. For this purpose, control and EAE mice were treated with and without 1.5% l-carnosine in drinking water (from 7 days before immunization). Carnosine treatment significantly increased spinal cord carnosine levels in both healthy and EAE mice (Fig. 4a). Notably, the magnitude of carnosine increase was strongly influenced by the EAE disease stage, with acute EAE mice displaying the greatest effect (absolute increase of 1.68 ± 0.49 nmol/mgWW) despite a shorter total treatment duration than the subacute and chronic EAE mice. In carnosine-treated control and chronic EAE mice, which displayed the smallest effect, substantial carnosine loading was still apparent in spinal cord (change compared to untreated reference group: control + 175%, acute EAE + 1363%, subacute EAE + 481%, chronic EAE + 126%). To determine whether increasing carnosine availability could affect the conjugation or removal of reactive carbonyls, we also examined the levels of the carnosine-carbonyl conjugates and found that the formation of carnosine-propanal within the spinal cord was significantly increased in a similar time-dependent manner (compared to untreated reference group: control + 23%, acute EAE + 268%, subacute EAE + 103%, chronic EAE + 36%, Fig. 4b). In addition, small levels of carnosine-propanol (the reduced carnosine-acrolein adduct) were detectable in mice receiving oral carnosine, but could not be quantified. Homocarnosine was slightly reduced by carnosine treatment (Fig. 4c). Long-term (28 and 56 days) carnosine treatment significantly increased anserine levels in EAE spinal cord (Fig. 4d). In conclusion, these findings show that exogenous carnosine abundantly reaches the spinal cord to enhance local carnosine levels and promote acrolein quenching. In EAE, these effects are most pronounced in the acute stage.

### Carnosine treatment dose-dependently attenuates EAE disease severity

**Fig. 5:**
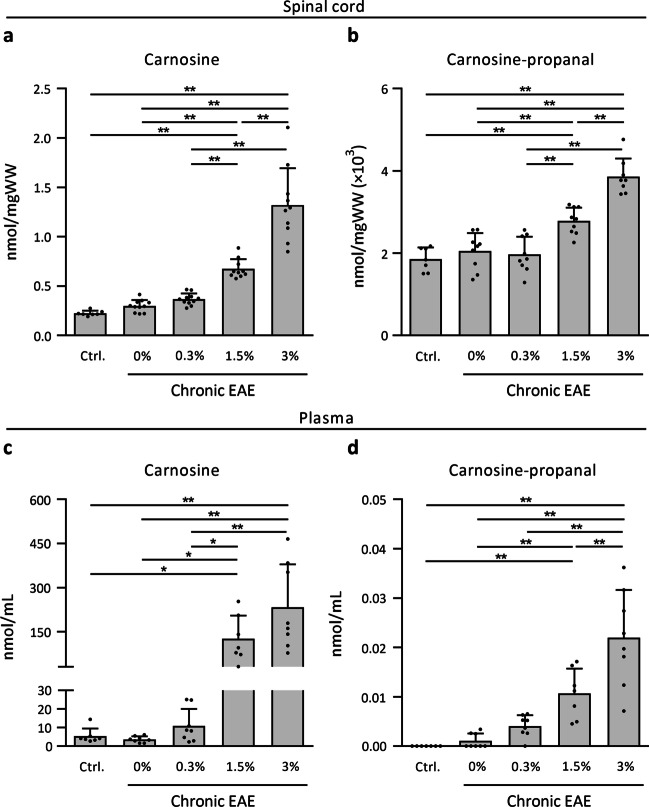
Carnosine treatment dose-dependently increases carnosine and carnosine-propanal in chronic EAE. UPLC-ESI–MS/MS-based quantification of spinal cord and plasma **a**, **c** carnosine and **b**, **d** carnosine-propanal in healthy controls, untreated EAE animals (0%) and following treatment with different doses of carnosine in drinking water (0.3%, 1.5%, 3%) in EAE. In figure **d**, carnosine-propanal was not detected in 7/7 control mice, 5/8 untreated EAE mice, and 1/8 mice receiving 0.3% carnosine. n = 7–11 animals/group. Carnosine-propanal values (~ 0.0015 nmol/mgWW) were transformed (× 10^3^) to facilitate visualisation on the Y axis. Data are mean ± SD. One-way ANOVA or Kruskal–Wallis, post hoc testing *p < 0.05, **p < 0.01 between the indicated groups

To investigate the therapeutic potential of carnosine treatment and whether carnosine enhances acrolein quenching in EAE, mice were treated with 3 different carnosine doses (0.3%, 1.5% or 3% in drinking water) and scored daily on a 0–5 scale for paresis assessment. To ensure dose-dependent carnosine loading, carnosine levels within the spinal cord isolated from the chronic stage were analysed. Compared to untreated EAE mice (0.30 ± 0.06 nmol/mgWW), carnosine levels were increased in the groups receiving 1.5% carnosine (0.68 ± 0.10 nmol/mgWW, + 126%) and 3% carnosine (1.32 ± 0.38 nmol/mgWW, + 343%, Fig. 5a). As expected, increased carnosine availability also increased acrolein quenching, with carnosine-propanal displaying a similar dose-dependent pattern in the 1.5% and 3% carnosine groups (0.0028 ± 0.0003 nmol/mgWW, + 36% and 0.0039 ± 0.0004 nmol/mgWW, + 89%, respectively) compared to untreated EAE mice (0.0020 ± 0.0004 nmol/mgWW, Fig. 5b). Carnosine-propanol was detectable, yet not quantifiable, in spinal cord of EAE mice receiving either 1.5% or 3% carnosine. Dose-dependent increases in carnosine and carnosine-propanal were also observed in plasma (Fig. 5c, d). Homocarnosine, anserine and GSH levels are presented in Additional file 4: Figure S5.

**Fig. 6:**
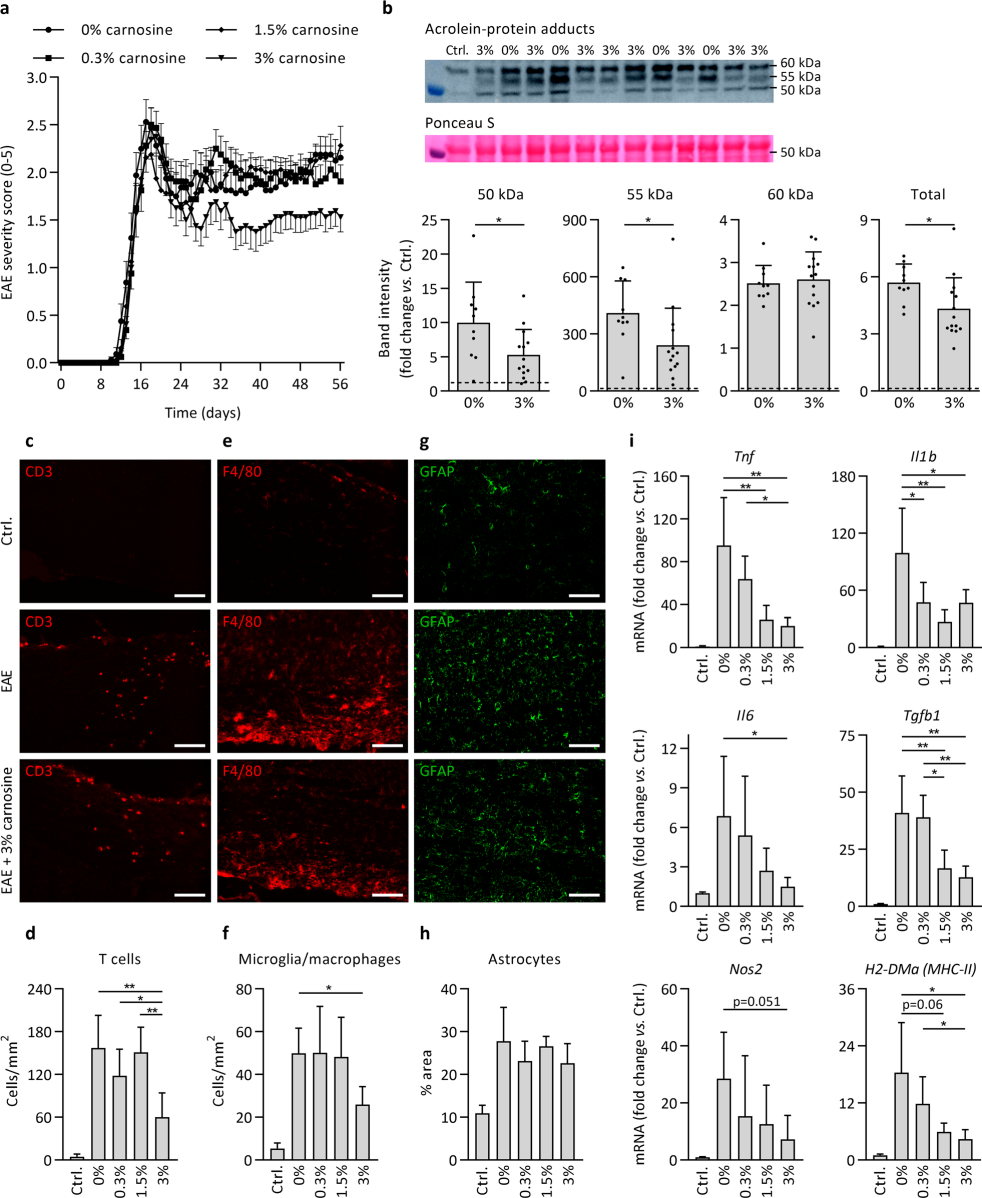
Carnosine treatment attenuates disease burden, acrolein-protein adduct formation and markers of neuroinflammation in EAE. **a** Clinical EAE disease severity, scored daily (day 0–56 post immunization) by a blinded assessor, indicates a significantly lower cumulative disease burden in animals receiving 3% carnosine compared to all other groups. n = 16 animals/group. **b** Detection of acrolein-protein adducts by western blot in healthy controls (dashed line), untreated EAE animals (0%) and following treatment with carnosine (3% in drinking water) in EAE. Groups receiving 0.3% and 1.5% were omitted to facilitate direct comparison of 0% and 3% carnosine on a limited number of blots. All treatment groups were compared in a preliminary experiment (data not shown). **c**, **d** Immunohistochemistry-based quantification of spinal cord T cell numbers (CD3) shows a reduction in carnosine-treated mice (3%) compared to untreated mice (0%) and mice treated with 0.3% or 1.5% carnosine in the drinking water. **e**, **f** Immunohistochemistry-based quantification of spinal cord microglia/macrophage numbers (F4/80) shows a reduction in carnosine-treated mice (3%) compared to untreated mice (0%). **g, h** Immunohistochemistry-based quantification of spinal cord reactive astrocyte area (GFAP) showed no treatment effect of carnosine. For figures **d**, **f** and **h**, all EAE groups exhibited a significant increase compared to healthy controls (statistics not shown). Figures **d**, **f**, **h**: n = 5–6 animals/group. **i** Spinal cord mRNA expression in healthy controls, untreated EAE animals (0%) and following treatment with different doses of carnosine (0.3%, 1.5%, 3% in the drinking water) in EAE. n = 5–6 animals/group. For all genes, untreated EAE groups (0%) exhibited a significant increase compared to controls (statistics not shown). Data are mean ± SD. One-way ANOVA or Kruskal–Wallis, post hoc testing *p < 0.05, **p < 0.01 between the indicated groups (**a**, **d**, **f**, **h**, **i**). Independent samples t-test *p < 0.05, control line is for reference (**b**). Scale bars are 100 µm (**c**, **e**, **g**)

EAE disease severity was attenuated by carnosine treatment. The clinical effect mainly occurred after the acute stage (Fig. 6a), and the cumulative clinical disability (area under the curve, AUC) was significantly lower in mice receiving 3% carnosine (AUC 68.9 ± 13.7) compared to all other groups (EAE, 85.0 ± 14.4; EAE 0.3%, 84.0 ± 14.4; EAE 1.5%, 82.9 ± 15.1; all p < 0.05). No clinical effect occurred at disease onset or peak. Mice were sacrificed 56 days post immunization. By western blot, we defined whether carnosine treatment was able to limit acrolein-protein adduct formation. As shown in Fig. 6b, carnosine treatment markedly reduced acrolein-protein adduct levels, by ~ 50% for proteins of 50 and 55 kDa and by ~ 25% overall. In contrast, HNE-protein adduct levels were unaffected by EAE and carnosine (Additional file 4: Fig. S6). The beneficial effect of carnosine on EAE disease severity was confirmed by immunohistochemistry showing reduced CD3^+^ T cell and F4/80^+^ microglia and macrophage numbers in spinal cord (Fig. 6c–f). Carnosine did not impact GFAP^+^ reactive astrocytes in spinal cord (Fig. 6g, h). Gene expression analysis revealed a dose-dependent reduction in inflammatory cytokines, *Nos2* and *H2-DMa* (MHC-II) mRNA levels following carnosine treatment (Fig. 6i). Finally, we aimed to replicate the protective effect of oral carnosine treatment (1.5% in drinking water) in a different species (rat) and EAE model (monophasic spontaneously recovering disease course, 17 days), and we could confirm a 30% reduction (p < 0.01) in EAE cumulative clinical disability (see details in Additional file 2: Table S2). Taken together, carnosine treatment dose-dependently attenuated clinical disease severity and markers of neuroinflammation in EAE. The effects are accompanied by increased acrolein quenching and reduced acrolein-protein adduct formation in spinal cord using high-dose carnosine treatment.

### Carnosine quenches inflammation-induced acrolein and modulates microglia and macrophage inflammation in vitro

**Fig. 7:**
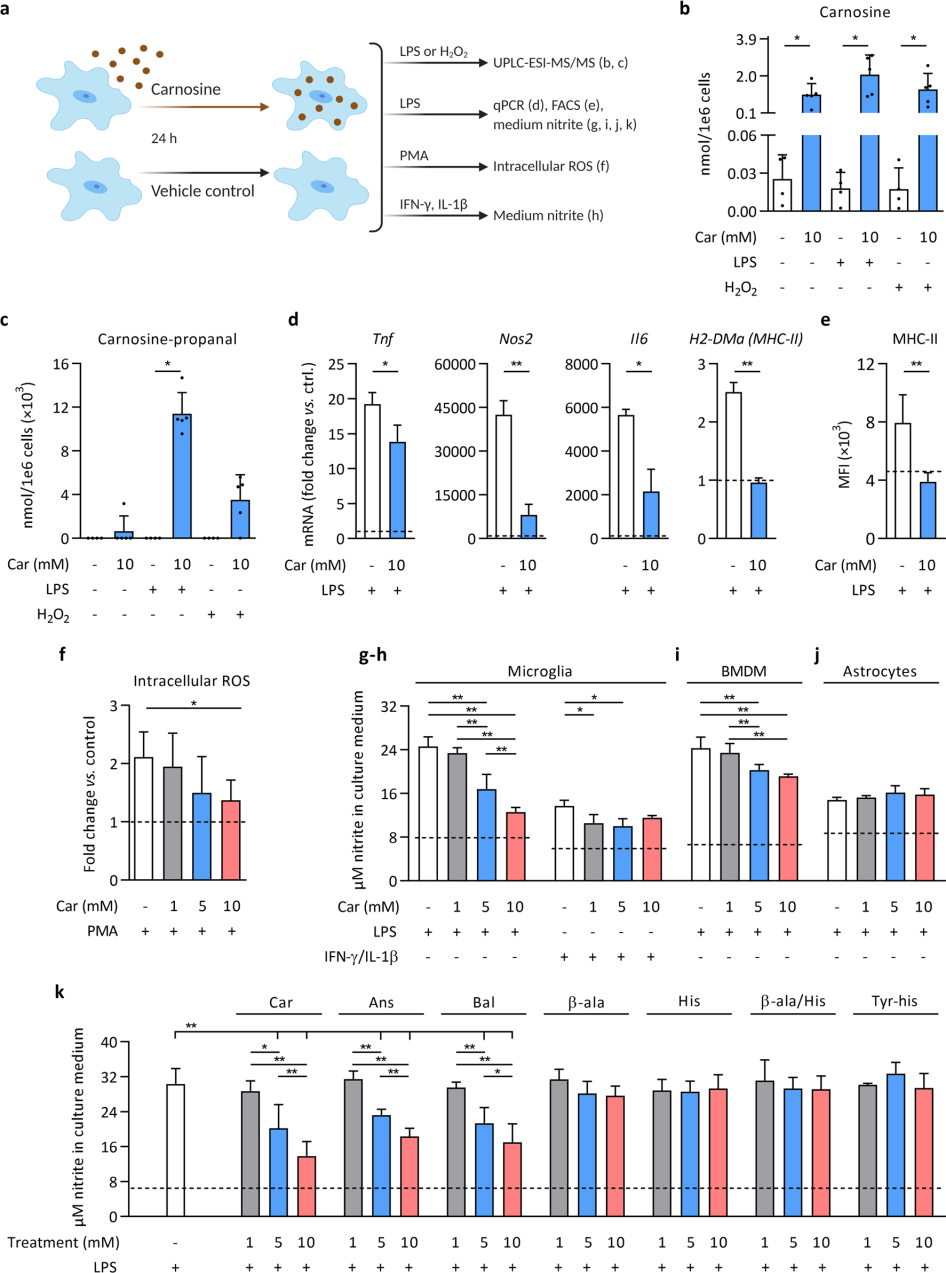
Carnosine quenches inflammation-induced acrolein and modulates microglia and macrophage inflammation in vitro*.* The experimental setup is shown in (**a**). For all experiments, cells were pre-treated with carnosine (or an alternative) for 24 h, after which the culture medium was refreshed and cells were exposed to LPS, H_2_O_2_, PMA, IFN-γ/IL-1β or vehicle control (PBS, represented by the horizontal dashed lines in **d**–**k**). **b**, **c** UPLC-ESI–MS/MS-based quantification of carnosine and carnosine-propanal in primary mouse microglia. n = 4–5 biological replicates/condition. Carnosine-propanal was undetectable in all experimental conditions lacking carnosine pre-treatment, in 4/5 biological replicates without LPS or H_2_O_2_ (following carnosine), and in 1/5 biological replicates using H_2_O_2_ (following carnosine). Carnosine-propanal values (~ 0.004 to 0.012 nmol/1e6 cells) were transformed (× 10^3^) to facilitate visualisation on the Y axis. **d** Gene expression in primary mouse microglia. n = 3 biological replicates/condition. **e** MHC-II mean fluorescence intensity (MFI) determined by flow cytometry on F4/80^+^ primary mouse microglia. n = 11 biological replicates/condition. **f** Intracellular ROS levels derived from DCFH-DA fluorescence in primary mouse microglia. n = 6 biological replicates/condition. **g**, **h** Nitrite concentration in the culture medium of primary mouse microglia following exposure to LPS or IFN-γ/IL-1β. n = 5–8 biological replicates/condition. **i** Nitrite concentration in the culture medium of primary mouse BMDMs and **j** primary mouse astrocytes**.** n = 5–8 biological replicates/condition. **k** Nitrite concentration in the culture medium of primary mouse microglia. Carnosine (Car), anserine (Ans), balenine (Bal), β-alanine (β-ala), histidine (His), β-alanine and histidine (β-ala/His), tyrosyl-histidine (Tyr-his). n = 5–13 biological replicates/condition. BMDM, bone marrow-derived macrophages; DCFH-DA, 2′,7′-Dichlorofluorescin diacetate; H_2_O_2_, hydrogen peroxide; LPS, lipopolysaccharide; MFI, mean fluorescence intensity; PBS, phosphate-buffered saline; PMA, phorbol 12-myristate 13-acetate; ROS, reactive oxygen species. Data are mean ± SD. Mann–Whitney U test with Bonferroni-adjusted p values for multiple testing *p < 0.05 (**b**, **c**). Mann–Whitney U test *p < 0.05, **p < 0.01 (**d**, **e**). One-way ANOVA or Kruskal–Wallis, post hoc testing *p < 0.05, **p < 0.01 between the indicated groups (**f**–**k**)

Given that acrolein generation in MS and EAE lesions spatially and temporally coincided with pro-inflammatory microglia/macrophages, and that acrolein quenching by carnosine suppressed neuroinflammation in EAE, we next used primary mouse microglial cell cultures to study acrolein generation and a potential direct anti-inflammatory effect of carnosine (Fig. 7a). First, UPLC-ESI–MS/MS analyses were performed to assess carnosine levels and acrolein quenching in microglia after exposure to carnosine (10 mM, 24 h) followed by LPS or H_2_O_2_ treatment. Microglia contained low basal levels of carnosine (~ 0.025 nmol/1e6 cells), which rose approximately 50- to 100-fold upon carnosine exposure (Fig. 7b). Interestingly, carnosine-propanal adducts were detected in carnosine-treated microglia under inflammatory conditions (TLR4 activation by LPS) or, to a lesser extent, following direct exposure to an oxidant (H_2_O_2_); illustrating both the acrolein-generating ability of inflammatory microglia and the quenching by carnosine (Fig. 7c). Carnosine and carnosine-propanal correlated with each other (Additional file 4: Fig. S7a), and when expressed per mg protein, carnosine and carnosine-propanal levels were within the same range in vitro (microglia) and in vivo (EAE spinal cord) (data not shown). Furthermore, low levels of anserine were observed in carnosine-treated cells (Additional file 4: Fig. S7b), whilst no homocarnosine, carnosine-propanol or carnosine-HNE was present. Cell viability was unaffected under all experimental conditions (Additional file 4: Fig. S7c).

To study if carnosine treatment directly affects inflammatory activity of microglia, microglial cultures were pre-treated with carnosine (10 mM, 24 h), exposed to an inflammatory trigger, and subsequently analysed for several functional/inflammatory indicators. We observed that carnosine suppressed gene expression of pro-inflammatory cytokines, *Nos2* and *H2-DMa* (MHC-II) following LPS exposure for 24 h (Fig. 7d) but not 6 h (data not shown). Consistent with these findings, flow cytometric analyses demonstrated that carnosine completely suppressed the LPS-induced MHC-II surface expression (Fig. 7e). Similarly, carnosine pre-treatment dose-dependently reduced microglial ROS levels (Fig. 7f) and nitrite release, an indicator of nitric oxide production (Fig. 7g). Carnosine did not affect basal nitrite release by naive microglia (data not shown). Suppressed nitrite release was confirmed, even at lower doses of carnosine, when using IFN-γ/IL-1β to activate microglia (Fig. 7h). We next investigated different cell populations relevant to neuroinflammation in MS/EAE, and found that carnosine also reduced nitrite release from mouse BMDMs (Fig. 7i), but not primary mouse astrocytes (Fig. 7j), suggesting a greater involvement of microglia/macrophages compared to astrocytes in the therapeutic effect of carnosine. Given that carnosine is a multifunctional dipeptide [17] and to examine whether the acrolein quenching ability of carnosine alleviates neuroinflammation, we next compared the effect of the dipeptide carnosine to its constituent amino acids (β-alanine, histidine, or both), its Nπ- and Nτ-methylated analogues (anserine, balenine) and an equimolar negative control (tyrosyl-histidine) on inflammation-induced cellular nitrite release by microglia. Carnosine and anserine have similar carbonyl quenching properties, whereas balenine, β-alanine and histidine exhibit low carbonyl quenching ability [21, 39]. As shown in Fig. 7k, pre-treatment with the dipeptides carnosine, anserine and balenine reduced nitrite release, in contrast to individual amino acids and negative control. At a concentration of 10 mM, carnosine was significantly more effective than anserine and balenine (p < 0.01). In summary, in vitro experiments indicate that inflammatory microglia/macrophages are a source of acrolein, which can be efficiently quenched by increasing carnosine availability, resulting in suppressed inflammatory activity. Yet, considering that acrolein quenching by balenine is minimal compared to carnosine and anserine, we cannot exclude that other properties, such as direct antioxidant activity or nitric oxide scavenging, also contribute to the versatile actions of carnosine (treatment) during neuroinflammation.

## Discussion

Whilst current MS therapeutics effectively target peripheral immune responses and/or the entrance of autoreactive lymphocytes into the CNS, there is an unmet need for local neuroprotective agents able to slow down the disease progression that accompanies sustained CNS inflammation and cellular injury [2, 4]. Acrolein, a highly reactive and toxic carbonyl, was strongly upregulated in inflammatory MS and EAE lesions, whereas the endogenous acrolein quencher carnosine was decreased in EAE. When tissue carnosine levels were increased via oral administration, acrolein-protein adduct formation and neuroinflammation were profoundly attenuated. In vivo and in vitro experiments suggest an essential role of microglia/macrophages in the generation of acrolein and the therapeutic effect of carnosine. Our findings identify carbonyl (in particular acrolein) quenching by carnosine as a therapeutic strategy to counter inflammation and macromolecular damage in MS, and possibly other neuroinflammatory disorders.

Among the variety of reactive carbonyls generated by oxidative decomposition of PUFAs, the 3-carbon α,β-unsaturated aldehyde acrolein is the strongest electrophile, capable of exerting long-lasting cytotoxic changes [6, 7, 40]. Recently, an increase in urinary and serum 3-hydroxypropyl mercapturic acid (3-HPMA), a GSH-acrolein metabolite, was reported in MS patients; for the first time proposing the pathological role of acrolein in MS [12]. In the present study, we have identified acrolein in active MS lesions. In EAE mice, acrolein-protein adduct formation in spinal cord appeared as a dynamic process related to disease stage and inflammatory activity. Likewise, acrolein-protein adducts in MS seemed more common in active than mixed active/inactive lesions. Acrolein damage in MS/EAE lesions occurred in a variety of structures in the brain parenchyma and perivascular spaces, often in close vicinity to microglia/macrophages and reactive astrocytes. Together with our in vitro findings that indicate acrolein generation by pro-inflammatory microglia, this implies that the generation of acrolein, pro-inflammatory activity and the oxidative burst are entwined and may occur in a self-sustaining manner during neuroinflammation. In fact, previous studies showed that low levels of acrolein can promote inflammatory activity and ROS production without affecting cell viability [41–43]. In addition, acrolein possesses strong cytotoxic effects through binding with essential cellular macromolecules, as evidenced by the great levels of acrolein-protein adducts in the present report. Interestingly, the strong increase in acrolein-protein adducts was not accompanied by the presence of HNE-protein adducts in EAE. Although we did not define specific molecular structures that are affected by acrolein and whether acrolein toxicity contributed directly to demyelination/oligodendropathy or axonal degeneration in MS [44], acrolein seems a valuable therapeutic target. Future research is warranted to decipher how, at what concentration, and in what disease stage(s) acrolein affects the (neuro)immune and degenerative processes that drive MS progression.

Given that reducing acrolein-protein adduct formation by carnosine was associated with attenuated disease severity in the EAE model further supports a pathological role of acrolein in MS. Carnosine quenched inflammation-induced acrolein and suppressed inflammatory and pro-oxidative activity in vitro and in vivo. Our results propose microglia/macrophages as important players in both the generation of acrolein and the therapeutic benefits of carnosine. The downstream effects of reducing acrolein levels in the CNS remain speculative but can be widespread. For example, by reducing cytokine and MHC-II expression, carnosine may indirectly affect T cell (re)activation or survival in the CNS [45]. A direct peripheral immunosuppressive effect of carnosine is less likely given the lack of effect at EAE disease onset and during development of the initial disease peak. It should be noted, however, that acrolein quenching may not explain all therapeutic benefits of carnosine. As can be expected from this versatile molecule, other properties including direct antioxidant or nitric oxide scavenging activity may contribute to its beneficial effects in EAE. This is particularly true given that at least some in vitro anti-inflammatory effects were retained by balenine, a methylated carnosine analogue with limited acrolein quenching ability [21, 39]. Finally, it should be noted that oral carnosine treatment was given prophylactically in order to allow sufficient time for tissue carnosine loading to occur. Future research should investigate in more depth how the timing of carnosine treatment affects the results.

Carbonyl quenching is a relatively novel therapeutic approach for MS, in contrast to traditional antioxidant therapies that often had limited clinical efficacy [46–48]. Previous efforts include the use of the carbonyl quenchers pyridoxamine and hydralazine in EAE. Pyridoxamine showed no beneficial effect in EAE [49]. Compared to carnosine, pyridoxamine is a less efficient acrolein quencher but more reactive towards malondialdehyde [16], suggesting that acrolein plays an important role in EAE pathogenesis and that the quenching agent needs to be chosen accordingly. Hydralazine is a very potent carbonyl quencher that improved EAE outcomes [11], even to a greater extent than observed for carnosine in the present study. However, hydralazine is a potent hypotensive drug that lacks quenching specificity and also reacts with physiological carbonyls, making it prone to elicit undesirable side-effects [16]. Since carnosine is an endogenous and food-derived molecule that has already been applied safely in human trials [29, 50] and even in a recent case series with three MS patients [51], larger-scale testing of carnosine intervention in MS may advance relatively quickly. Despite carnosine’s favorable toxicological profile and ability to cross the blood–brain barrier, one major disadvantage is a limited stability in human plasma due to the presence of the carnosine-degrading enzyme serum carnosinase or CN1 (not present in rodents) [17, 52]. Although the CNS possesses carnosine synthase activity to re-synthesize carnosine from β-alanine and histidine, it may be worthwhile to consider carnosinase-resistant carnosine analogues for application in humans; especially since carbonyl quenching properties of carnosine are diminished or lost by the individual amino acids. For example, the recently developed synthetic carnosine analogue carnosinol maintains physiological properties but is resistant to degradation [8, 53, 54]. The natural carnosine analogue anserine also has better stability towards CN1 and retains acrolein-quenching ability [55].

Tissue carnosine homeostasis is maintained by a complex interplay between enzymatic synthesis and hydrolysis reactions, amino acid/dipeptide transport, and conjugation with reactive carbonyls such as acrolein [17, 20]. In the present study we found that the levels of endogenous carnosine and carnosine-acrolein conjugates were depleted and the levels of acrolein-protein adducts were increased within the spinal cord of EAE mice. Given that carnosine binds with acrolein, increased sacrificial acrolein quenching and extrusion of carnosine-acrolein conjugates might contribute to the depleted carnosine pool (− 46%). Although homocarnosine (in which β-alanine is replaced by γ-aminobutyric acid [GABA]) was the most abundant HCD present in the mouse spinal cord, only carnosine showed detectable quenching activity towards acrolein, forming carnosine-propanal. In addition, mRNA data suggests that decreased synthesis (*Carns1*) and/or increased breakdown (*Cndp1/2*) could also contribute towards carnosine depletion in EAE spinal cord. In the mouse CNS, *Cndp2* (as well as transporters *Pht1/2*, *Pept2*) are mainly expressed by microglia and astrocytes [56], which may explain their general increase during EAE. Human and mouse RNA sequencing studies indicate that *CARNS1* is almost exclusively expressed in mature oligodendrocytes [56–63]. Oligodendrocyte dysfunction, death and demyelination may therefore underlie the reduction in *Carns1* expression seen in acute EAE [64]. In contrast, by single-nucleus RNA sequencing of human brain white matter, Jäkel et al*.* found that *CARNS1* expression was increased in active MS lesions compared to healthy controls [65]. As we found that *Carns1* expression was restored in later stages of EAE, along with gradually rising tissue carnosine levels (even slightly higher than control mice), this may indicate a protective response. A similar mechanism has been suggested for muscle tissue of type 2 diabetes mellitus patients, where elevated carnosine levels presumably reflect a higher need for defence against oxidative and carbonyl stress [66]. To fully understand the significance of carnosine homeostasis in CNS health and disease, additional research is strongly recommended.

It is worth emphasizing the remarkable ability to increase carnosine levels in the mouse spinal cord upon oral carnosine intake. In fact, spinal cord seemed to have an overall greater increase in carnosine compared to muscle tissue, which has been extensively studied and is already well-known for its ability to augment carnosine content [17]. To allow direct comparison between spinal cord and muscle tissue, we assessed carnosine levels in *m.* tibialis anterior from healthy and EAE mice by UPLC-ESI–MS/MS. Compared to muscle, the spinal cord of healthy mice exhibited a greater absolute (nmol/mgWW, ~ 1.4-fold greater increase) and especially relative increase (%, ~ sixfold greater increase). Data are available upon request and were consistent with previous reports of muscle carnosine loading, e.g. [67]. In EAE mice, the amount of carnosine loading was both dose- and time-dependent. The fact that carnosine loading efficiency was enhanced during the (sub)acute stages of disease, reaching carnosine levels > tenfold of baseline, further highlights that the regulation of carnosine homeostasis is sensitive to disease-associated alterations. Although hypothetical, this may be explained by a local hypercellular environment featured by an abundance of dipeptide transporters on microglia/macrophages and astrocytes. In addition, it was previously shown that inflammation (LPS/IFN-γ exposure) can stimulate carnosine uptake in RAW 264.7 macrophages by threefold [68]. Altogether, this illustrates a previously unknown capacity of the spinal cord to take up and retain carnosine, and underscores the favorable bioavailability of oral carnosine. Finally, the fact that anserine levels increased with augmented carnosine availability suggests the presence of an active protein methyltransferase enzyme, probably carnosine *N*-methyltransferase (CARNMT1); whilst the detection of carnosine-propanol in carnosine-treated EAE mice implies the reduction of carnosine-propanal by aldose reductase (AR) in spinal cord [20, 69].

## Conclusion

To conclude, increased acrolein generation and the resulting macromolecular damage may play a crucial pathological role during neuroinflammation in MS and EAE. This was associated with depletion of carnosine, an endogenous acrolein quencher. Oral carnosine treatment prevented acrolein damage in EAE spinal cord and attenuated neuroinflammation and diseases severity. Our findings pave the way for future research focusing on the effect of carbonyl (in particular acrolein) quenching in neuroinflammatory disorders such as MS. In addition, we highlight the need for a better understanding of endogenous carnosine homeostasis in the CNS.

## Supplementary Information

**Additional file 1: Table S1.** Clinical details of human brain tissue.

**Additional file 2: Table S2.** Oral carnosine treatment attenuates clinical disability in a monophasic rat EAE model.

**Additional file 3: Table S3.** Primer sequences.

**Additional file 4: Figure S1.** Graphical summary of experimental designs. Graphical representation of the timing of carnosine treatment, EAE induction and sacrifice (tissue collection) for (a) analyses of control vs. EAE mice (data shown in Figure 2, Figure 3, Figure S2, Figure S3, Figure S4); (b) analysis of tissue carnosine loading in EAE (data shown in Figure 4); and (c) analyses of dose-dependent carnosine treatment effects (data shown in Figure 5, Figure 6, Figure S5, Figure S6). **Figure S2.** Spinal cord inflammatory activity during EAE. (a) Spinal cord mRNA expression in different stages of EAE compared to healthy controls (dashed line). n=6 animals/group. (b) Immunohistochemistry-based quantification of F4/80^+^ microglia/macrophage numbers in the spinal cord in different stages of EAE compared to healthy controls (dashed line). n=6 animals/group. Gene expression (a) and microglia/macrophage numbers (b) were increased in EAE vs. healthy controls (statistics not shown). Data are mean ± SD. One-way ANOVA or Kruskal-Wallis, post hoc testing *p<0.05, **p<0.01 between the indicated groups. **Figure S3.** HNE-protein adducts are not affected by EAE. Detection of HNE-protein adducts by western blot in different stages of EAE compared to healthy controls. n=5-7 animals/group. Data are mean ± SD. One-way ANOVA indicated no significant between-group differences. **Figure S4.** Spinal cord GSH levels in EAE. UPLC-ESI-MS/MS-based quantification of spinal cord GSH in different stages of EAE compared to healthy controls. n=11-22 animals/group. GSH, reduced glutathione. Data are mean ± SD. One-way ANOVA, post hoc testing *p<0.05, **p<0.01 between the indicated groups. **Figure S5.** Effects of carnosine treatment on homocarnosine, anserine and GSH levels in chronic EAE. UPLC-ESI-MS/MS-based quantification of spinal cord and plasma (a, d) homocarnosine, (b, e) anserine and (c, f) GSH in healthy controls, untreated EAE animals (0%) and following treatment with different doses of carnosine (0.3%, 1.5%, 3%) in EAE. n=7-11 animals/group. Data are mean ± SD. One-way ANOVA or Kruskal-Wallis, post hoc testing *p<0.05, **p<0.01 between the indicated groups. **Figure S6.** No effect of EAE and carnosine treatment on HNE-protein adduct levels. Detection of HNE-protein adducts by western blot in healthy controls, untreated EAE animals (0%) and following treatment with different doses of carnosine (0.3%, 1.5%, 3% in drinking water) in EAE. n=7-15 animals/group. Data are mean ± SD. One-way ANOVA indicated no significant between-group differences. **Figure S7.** Effect of carnosine on acrolein quenching, anserine levels and cell viability in primary mouse microglia cultures. UPLC-ESI-MS/MS-based quantification of (a) carnosine and carnosine-propanal and (b) anserine in primary mouse microglia exposed to carnosine or vehicle control (PBS) for 24 h, followed by LPS, H_2_O_2_ or vehicle control (PBS) for 24 h. n=4-5 biological replicates/condition. Carnosine-propanal values (~0.004 to 0.012 nmol/1e6 cells) were transformed (×10^3^) to facilitate visualisation on the Y axis. Anserine was undetectable in all experimental conditions lacking carnosine pre-treatment. (c) Cell viability of primary microglia assessed by flow cytometry (Fixable Viability Dye eFluor 506). Cells were exposed to carnosine or vehicle control (PBS) for 24 h, followed by LPS, H_2_O_2_ or vehicle control (PBS) for 24 h. Data from n=7 different experiments, normalized to the respective control condition in each experiment and expressed as % change. Car, carnosine. H_2_O_2_, hydrogen peroxide; LPS, lipopolysaccharide; PBS, phosphate-buffered saline. Data are mean ± SD. Mann-Whitney U test with Bonferroni-adjusted p values for multiple testing *p<0.05 (b). One-way ANOVA indicated no significant between-group differences (c).

## Data Availability

The datasets used and/or analysed during the current study are available through the corresponding author on reasonable request.

## Abbreviations

AUC: Area under the curve
BMDM: Bone marrow-derived macrophages
CN1: Serum carnosinase
CNS: Central nervous system
DAB: 3,3′-Diaminobenzidine
DCFH-DA: 2′,7′-Dichlorofluorescin diacetate
EAE: Experimental autoimmune encephalomyelitis
FVD: Fixable viability dye
GFAP: Glial fibrillary acidic protein
GSH: Glutathione
H_2_O_2_: Hydrogen peroxide
HCD: Histidine-containing dipeptide
HNE: 4-Hydroxy-2-nonenal
LCM: L929 cell-conditioned medium
LPS: Lipopolysaccharide
MS: Multiple sclerosis
PBS: Phosphate-buffered saline
PLP: Proteolipid protein
PMA: Phorbol 12-myristate 13-acetate
PUFA: Poly-unsaturated fatty acids
ROS: Reactive oxygen species
UPLC-ESI–MS/MS: Ultrahigh-performance liquid chromatography electrospray ionization tandem mass spectrometry
WW: Wet tissue weight
