# Supplementary material for: Carnosine quenches the reactive carbonyl acrolein in the central nervous system and attenuates autoimmune neuroinflammation
Source: J Neuroinflammation. 2021 Nov 5;18:255. doi: 10.1186/s12974-021-02306-9 (PMC8571880; doi:10.1186/s12974-021-02306-9)
Supplement: Supplementary file 1 — Additional file 1: Table S1. Clinical details of human brain tissue. [file 12974_2021_2306_MOESM1_ESM.docx]

**Supplementary Table S1. Clinical details of human brain tissue.**

| **Autopsy** | **Age (y)** | **Gender** | **Post-mortem delay** | **Region** | **MS type** |
| --- | --- | --- | --- | --- | --- |
| 03/142 (n=2 tissue blocks) | 53 | Male | 5 h, 30 min | Plaque not MRI-scanned | PPMS |
| 04/172 | 43 | Female | 10 h, 45 min | Plaque not MRI-scanned | SPMS |
| 11/080 | 56 | Female | 8 h, 25 min | Plaque MRI-scanned | PMS |
| 08/292 | 61 | Male | 9 h, 15 min | Plaque MRI-scanned | SPMS |
| 17/001 | 39 | Female | 8 h, 30 min | Plaque MRI-scanned | SPMS |
| 07/314 | 66 | Female | 6 h, 00 min | Plaque not MRI-scanned | SPMS |
| 11/077 | 66 | Female | 9 h, 35 min | Plaque MRI-scanned | PPMS |
| 16/104 | 49 | Female | 8 h, 30 min | Plaque MRI-scanned | PPMS |
| 15/017 | 54 | Female | 9 h, 25 min | NAWM | SPMS |
| 01/058 | 48 | Female | 8 h, 10 min | NAWM | * |
| 96/163 | 69 | Female | 8 h, 30 min | White matter | NDC |

NAWM, normal-appearing white matter; NDC, non-demented control; PMS, progressive multiple sclerosis, not specified; PPMS, primary progressive multiple sclerosis; SPMS, secondary progressive multiple sclerosis; *, no specified diagnosis of MS type: patient suffered from MS exacerbations in the past (presumably relapsing-remitting MS, the final documented exacerbation occurred 4 y before death), which may have transitioned into a secondary progressive phase.
