# Supplementary material for: Carnosine quenches the reactive carbonyl acrolein in the central nervous system and attenuates autoimmune neuroinflammation
Source: J Neuroinflammation. 2021 Nov 5;18:255. doi: 10.1186/s12974-021-02306-9 (PMC8571880; doi:10.1186/s12974-021-02306-9)
Supplement: Supplementary file 2 — Additional file 2: Table S2. Oral carnosine treatment attenuates clinical disability in a monophasic rat EAE model. [file 12974_2021_2306_MOESM2_ESM.docx]

**Supplementary Table S2. Oral carnosine treatment attenuates clinical disability in a monophasic rat EAE model.**

| **EAE clinical disability** | **Control** | **Carnosine** |
| --- | --- | --- |
| EAE incidence (n, %) | 13/13 (100%) | 13/13 (100%) |
| Survival (n, %) | 10/13 (77%) | 13/13 (100%) |
| Disease onset (days) | 9.8 ± 0.7 | 10.4 ± 0.5 * |
| Peak (score) | 3.8 ± 0.7 | 3.1 ± 0.3 ** |
| Peak (days) | 11.8 ± 0.4 | 12.5 ± 0.7 ** |
| Recovery (days) | 4.8 ± 0.9 | 3.2 ± 0.9 ** |
| Cumulative disease score | 14.4 ± 3.6 | 10.1 ± 2.0 ** |

Female Lewis rats (age 6-7 weeks, body weight 100-120 g, Harlan CPB) were immunized with subcutaneuous injections (2 × 100 µL) containing 24 µL purified myelin basic protein (MBP, 25 mg/mL), 25 µL 7RA heat-killed *Mycobacterium Tuberculosis* (20 mg/ml, Difco), 120 µL complete Freund’s adjuvant (CFA, Difco) and 31 µL phosphate-buffered saline (PBS). Rats developed a monophasic EAE disease course; peaking ~12 days after immunization followed by complete recovery by day 17. Rats received normal tap water or tap water supplemented with 1.5% (15 g/L) carnosine (n=13/group). Clinical disability was scored daily on a 0-5 scale (including 0.5 increments) by a blinded assessor: 0, no symptoms; 1, limp tail; 2, hindlimb paresis; 3, hindlimb paralysis; 4, hindlimb and trunk (up to diaphragm) paralysis; 5, death due to EAE. Rats were sacrificed 17 days following immunization. In case animals died during the experiment (score 5, n=3), no clinical scores were given in the subsequent days. *p<0.05, **p<0.01 using independent t-tests. Survival was analyzed by Fisher Exact test (p=0.22).
