# Supplementary material for: Carnosine quenches the reactive carbonyl acrolein in the central nervous system and attenuates autoimmune neuroinflammation
Source: J Neuroinflammation. 2021 Nov 5;18:255. doi: 10.1186/s12974-021-02306-9 (PMC8571880; doi:10.1186/s12974-021-02306-9)
Supplement: Supplementary file 3 — Additional file 3: Table S3. Primer sequences. [file 12974_2021_2306_MOESM3_ESM.docx]

**Supplementary Table S3. Primer sequences.**

| **Gene** | **Full name** | **Fw primer sequence** | **Rv primer sequence** |
| --- | --- | --- | --- |
| *Carns1* | carnosine synthase | TGATAGGCCCCTACTGAGTAAGGT | TCAGTGTCCTTGGCAGGGTAT |
| *Cndp1* | carnosine dipeptidase 1 | AGGAGTGGGTGGCCATTGA | CAAGGCCATCATCTGGAAGAG |
| *Cndp2* | cytosolic non-specific dipeptidase 2 | GGAGATACCACTTCCTCCCATTC | CGTCCAGGTGCCCGTAAAT |
| *H2-DMa* (MHC-II) | major histocompatibility complex class II, locus DMa | GCTCTCGGAGACCTATGACG | ACAGGCAAACCTCTGGACAC |
| *Il1b* | interleukin 1 beta | GCTGAAAGCTCTCCACCTCA | AGGCCACAGGTATTTTGTCG |
| *Il6* | interleukin 6 | CACTTCACAAGTCGGAGGCT | CTGCAAGTGCATCATCGTTGT |
| *Nos2* | nitric oxide synthase 2, inducible | CCCTTCAATGGTTGGTACATGG | ACATTGATCTCCGTGACAGCC |
| *Nrf2* | nuclear factor erythroid 2-related factor 2 | CGAGATATACGCAGGAGAGGTAAGA | GCTCGACAATGTTCTCCAGCTT |
| *Pept2* | peptide transporter 2 (Slc15a2) | TGGCTGGGAAAATTCAAGACA | ATGGCACCCAAAGACTTGAATAC |
| *Pht1* | peptide/histidine transporter 1 (Slc15a4) | CATGTGTCCGTGGTGATTGAG | GCGTGGTGTAACTGCCAATCT |
| *Pht2* | peptide/histidine transporter 2 (Slc15a3) | GCTGACCAGGTGATGGATCTC | AATATGGCACCCAGGTTGATG |
| *Taut* | taurine transporter (Slc6a6) | TGGCCGACAGCATTCCA | GCCTTCTCTAAGGTGCCTTCCT |
| *Tgfb1* | transforming growth factor beta 1 | GGGCTACCATGCCAACTTCTG | GAGGGCAAGGACCTTGCTGTA |
| *Tnf* | tumor necrosis factor | ATGGCCTCCCTCTCATCAGT | CTTGGTGGTTTGCTACGACG |
| **Housekeeping genes** | |  |  |
| *Actb* | beta actin | GGCTGTATTCCCCTCCATCG | CAGTTGGTAACAATGCCATGT |
| *Hmbs* | hydroxymethyl-bilane synthase | GAGACCATGCAGGCCACCAT | TTGGAATGTTCCGGGCAGTG |
| *Hprt* | hypoxanthine phosphoribosyltransferase 1 | CTCATGGACTGATTATGGACAGGAC | GCAGGTCAGCAAAGAACTTATAGCC |
| *Rpl13a* | ribosomal protein L 13a | GGATCCCTCCACCCTATGACA | CTGGTACTTCCACCCGACCTC |
| *Tbp* | TATA-box binding protein | ATGGTGTGCACAGGAGCCAAG | TCATAGCTACTGAACTGCTG |

List of mouse primers used for gene expression analysis (qPCR) of mouse spinal cord and primary microglia cell culture. A combination of at least 2 housekeeping genes was used for normalisation in different experiments, based on gene stability analysis with geNorm software.
