# Supplementary material for: Carnosine quenches the reactive carbonyl acrolein in the central nervous system and attenuates autoimmune neuroinflammation
Source: J Neuroinflammation. 2021 Nov 5;18:255. doi: 10.1186/s12974-021-02306-9 (PMC8571880; doi:10.1186/s12974-021-02306-9)
Supplement: Supplementary file 4 — Additional file 4: Figure S1. Graphical summary of experimental designs. Graphical representation of the timing of carnosine treatment, EAE induction and sacrifice (tissue collection) for (a) analyses of control vs. EAE mice (data shown in Figure 2, Figure 3, Figure S2, Figure S3, Figure S4); (b) analysis of tissue carnosine loading in EAE (data shown in Figure 4); and (c) analyses of dose-dependent carnosine treatment effects (data shown in Figure 5, Figure 6, Figure S5, Figure S6). Figure S2. Spinal cord inflammatory activity during EAE. (a) Spinal cord mRNA expression in different stages of EAE compared to healthy controls (dashed line). n=6 animals/group. (b) Immunohistochemistry-based quantification of F4/80+ microglia/macrophage numbers in the spinal cord in different stages of EAE compared to healthy controls (dashed line). n=6 animals/group. Gene expression (a) and microglia/macrophage numbers (b) were increased in EAE vs. healthy controls (statistics not shown). Data are mean ± SD. One-way ANOVA or Kruskal-Wallis, post hoc testing *p<0.05, **p<0.01 between the indicated groups. Figure S3. HNE-protein adducts are not affected by EAE. Detection of HNE-protein adducts by western blot in different stages of EAE compared to healthy controls. n=5-7 animals/group. Data are mean ± SD. One-way ANOVA indicated no significant between-group differences. Figure S4. Spinal cord GSH levels in EAE. UPLC-ESI-MS/MS-based quantification of spinal cord GSH in different stages of EAE compared to healthy controls. n=11-22 animals/group. GSH, reduced glutathione. Data are mean ± SD. One-way ANOVA, post hoc testing *p<0.05, **p<0.01 between the indicated groups. Figure S5. Effects of carnosine treatment on homocarnosine, anserine and GSH levels in chronic EAE. UPLC-ESI-MS/MS-based quantification of spinal cord and plasma (a, d) homocarnosine, (b, e) anserine and (c, f) GSH in healthy controls, untreated EAE animals (0%) and following treatment with different doses of carnosine (0. [file 12974_2021_2306_MOESM4_ESM.pdf]

SUPPLEMENTARY FIGURES

Carnosine quenches the reactive carbonyl acrolein in the central nervous system and attenuates autoimmune neuroinflammation

Jan Spaas[1,2,3], Wouter M. A. Franssen[2,4], Charly Keytsman[1,2,4], Laura Blancquaert[3], Tim Vanmierlo[1,5,6], Jeroen Bogie[1,2], Bieke Broux[1,5,7], Niels Hellings[1,5], Jack van Horssen[1,2,8], Dheeraj Kumar Posa[9], David Hoetker[9], Shahid P. Baba[9], Wim Derave[3,*], Bert O. Eijnde[1,2,*]

[1] University MS Center (UMSC) Hasselt – Pelt, Belgium.

[2] BIOMED Biomedical Research Institute, Faculty of Medicine and Life Sciences, Hasselt University, Hasselt, Belgium.

[3] Department of Movement and Sports Sciences, Faculty of Medicine and Health Sciences, Ghent University, Ghent, Belgium.

[4] REVAL Rehabilitation Research Center, Faculty of Rehabilitation Sciences, Hasselt University, Hasselt, Belgium.

[5] Neuro-Immune Connections and Repair Lab, Department of Immunology and Infection, Biomedical Research Institute, Hasselt University, Diepenbeek, Belgium.

[6] Department Psychiatry and Neuropsychology, Division of translational neuroscience, European Graduate School of Neuroscience, School for Mental Health and Neuroscience, Maastricht University, Maastricht, The Netherlands.

[7] Cardiovascular Research Institute Maastricht, Department of Internal Medicine, Maastricht University, Maastricht, The Netherlands.

[8] Department of Molecular Cell Biology and Immunology, Amsterdam Neuroscience, MS Center Amsterdam, Amsterdam University Medical Center, Location VUmc, Amsterdam, The Netherlands.

[9] Diabetes and Obesity Center, University of Louisville, Louisville, Kentucky, USA.

[*] equal contribution

Supplementary Figure S1. Graphical summary of experimental designs. Graphical representation of the timing of carnosine treatment, EAE induction and sacrifice (tissue collection) for **(a)** analyses of control *vs.* EAE mice (data shown in Figure 2, Figure 3, Figure S2, Figure S3, Figure S4); **(b)** analysis of tissue carnosine loading in EAE (data shown in Figure 4); and **(c)** analyses of dose-dependent carnosine treatment effects (data shown in Figure 5, Figure 6, Figure S5, Figure S6).

Supplementary Figure S2. Spinal cord inflammatory activity during EAE. (a) Spinal cord mRNA expression in different stages of EAE compared to healthy controls (dashed line). n=6 animals/group. **(b)** Immunohistochemistry-based quantification of F4/80^{+} microglia/macrophage numbers in the spinal cord in different stages of EAE compared to healthy controls (dashed line). n=6 animals/group. Gene expression (a) and microglia/macrophage numbers (b) were increased in EAE *vs.* healthy controls (statistics not shown). Data are mean ± SD. One-way ANOVA or Kruskal-Wallis, post hoc testing *p<0.05, **p<0.01 between the indicated groups.

Supplementary Figure S3. HNE-protein adducts are not affected by EAE. Detection of HNE-protein adducts by western blot in different stages of EAE compared to healthy controls. n=5-7 animals/group. Data are mean ± SD. One-way ANOVA indicated no significant between-group differences.

Supplementary Figure S4. Spinal cord GSH levels in EAE. UPLC-ESI-MS/MS-based quantification of spinal cord GSH in different stages of EAE compared to healthy controls. n=11-22 animals/group. GSH, reduced glutathione. Data are mean ± SD. One-way ANOVA, post hoc testing *p<0.05, **p<0.01 between the indicated groups.

Supplementary Figure S5. Effects of carnosine treatment on homocarnosine, anserine and GSH levels in chronic EAE. UPLC-ESI-MS/MS-based quantification of spinal cord and plasma **(a, d)** homocarnosine, **(b, e)** anserine and **(c, f)** GSH in healthy controls, untreated EAE animals (0%) and following treatment with

different doses of carnosine (0.3%, 1.5%, 3%) in EAE. n=7-11 animals/group. Data are mean ± SD. One-way ANOVA or Kruskal-Wallis, post hoc testing *p<0.05, **p<0.01 between the indicated groups.

Supplementary Figure S6. No effect of EAE and carnosine treatment on HNE-protein adduct levels. Detection of HNE-protein adducts by western blot in healthy controls, untreated EAE animals (0%) and following treatment with different doses of carnosine (0.3%, 1.5%, 3% in drinking water) in EAE. n=7-15 animals/group. Data are mean ± SD. One-way ANOVA indicated no significant between-group differences.

Supplementary Figure S7. Effect of carnosine on acrolein quenching, anserine levels and cell viability in primary mouse microglia cultures. UPLC-ESI-MS/MS-based quantification of **(a)** carnosine and carnosine-propanal and **(b)** anserine in primary mouse microglia exposed to carnosine or vehicle control (PBS) for 24 h, followed by LPS, H_2O_2 or vehicle control (PBS) for 24 h. n=4-5 biological replicates/condition. Carnosine-propanal values (~0.004 to 0.012 nmol/1e6 cells) were transformed ($\times 10^3$) to facilitate visualisation on the Y axis. Anserine was undetectable in all experimental conditions lacking carnosine pre-treatment. **(c)** Cell viability of primary microglia assessed by flow cytometry (Fixable Viability Dye eFluor 506). Cells were exposed to carnosine or vehicle control (PBS) for 24 h, followed by LPS, H_2O_2 or vehicle control (PBS) for 24 h. Data from n=7 different experiments, normalized to the respective control condition in each experiment and expressed as % change. Car, carnosine. H_2O_2, hydrogen peroxide; LPS, lipopolysaccharide; PBS, phosphate-buffered saline. Data are mean ± SD. Mann-Whitney U test with Bonferroni-adjusted p values for multiple testing *p<0.05 (b). One-way ANOVA indicated no significant between-group differences (c).

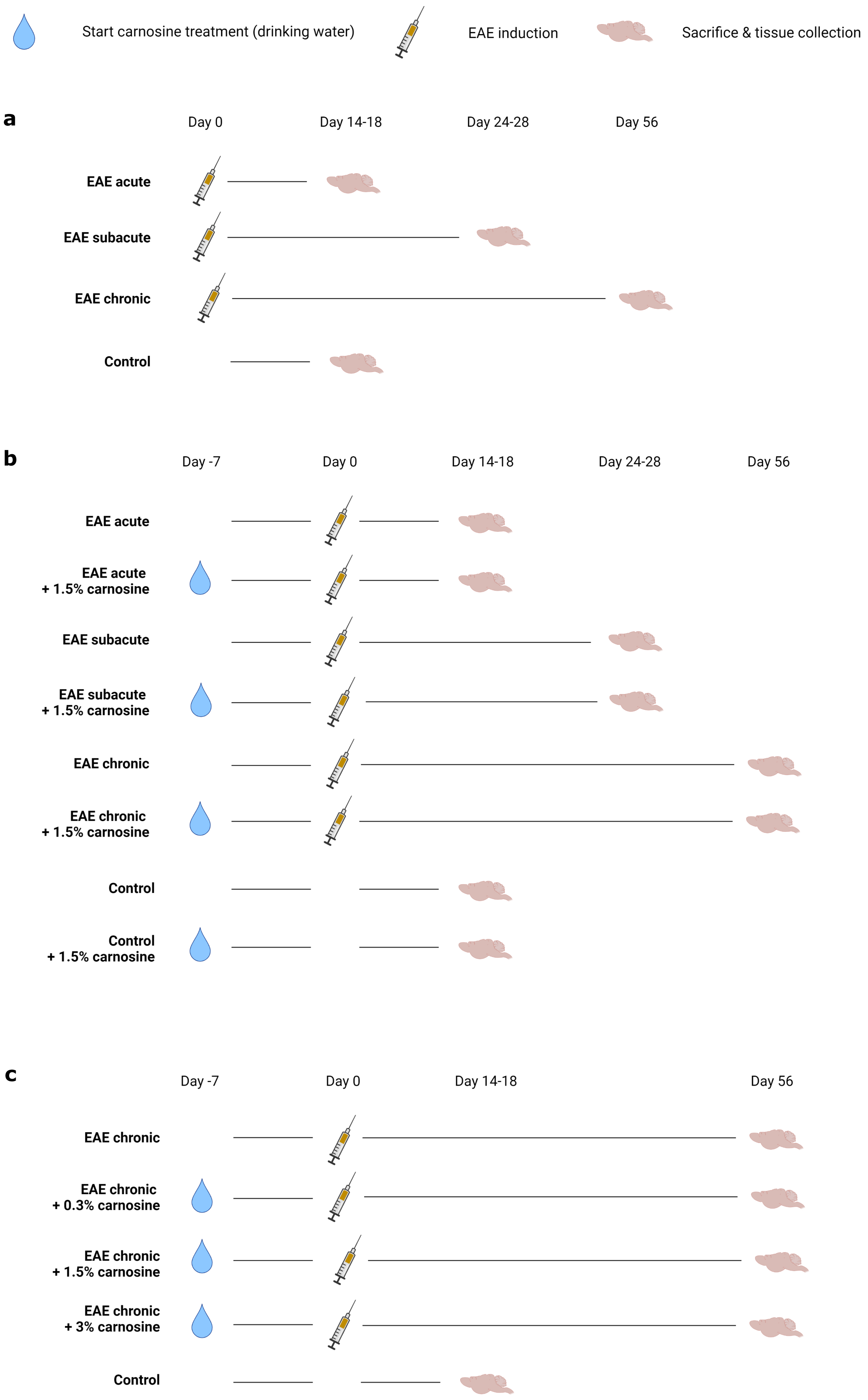
Start carnosine treatment (drinking water)
EAE induction
Sacrifice & tissue collection
a
Day 0
Day 14-18
Day 24-28
Day 56
EAE acute
EAE subacute
EAE chronic
Control
b
Day -7
Day 0
Day 14-18
Day 24-28
Day 56
EAE acute
EAE acute + 1.5% carnosine
EAE subacute
EAE subacute + 1.5% carnosine
EAE chronic
EAE chronic + 1.5% carnosine
Control
Control + 1.5% carnosine
c
Day -7
Day 0
Day 14-18
Day 56
EAE chronic
EAE chronic + 0.3% carnosine
EAE chronic + 1.5% carnosine
EAE chronic + 3% carnosine
Control

Figure S1

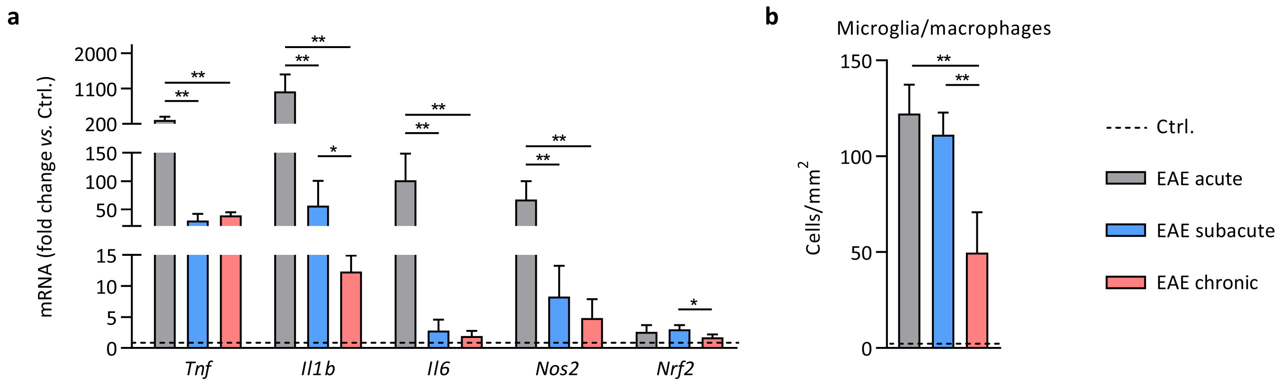

a
b
Microglia/macrophages
mRNA (fold change vs. Ctrl.)
2000
1100
200
150
100
50
15
10
5
0
Tnf
Il1b
Il6
Nos2
Nrf2
Cells/mm2
150
100
50
0
Ctrl.
EAE acute
EAE subacute
EAE chronic

Figure S2

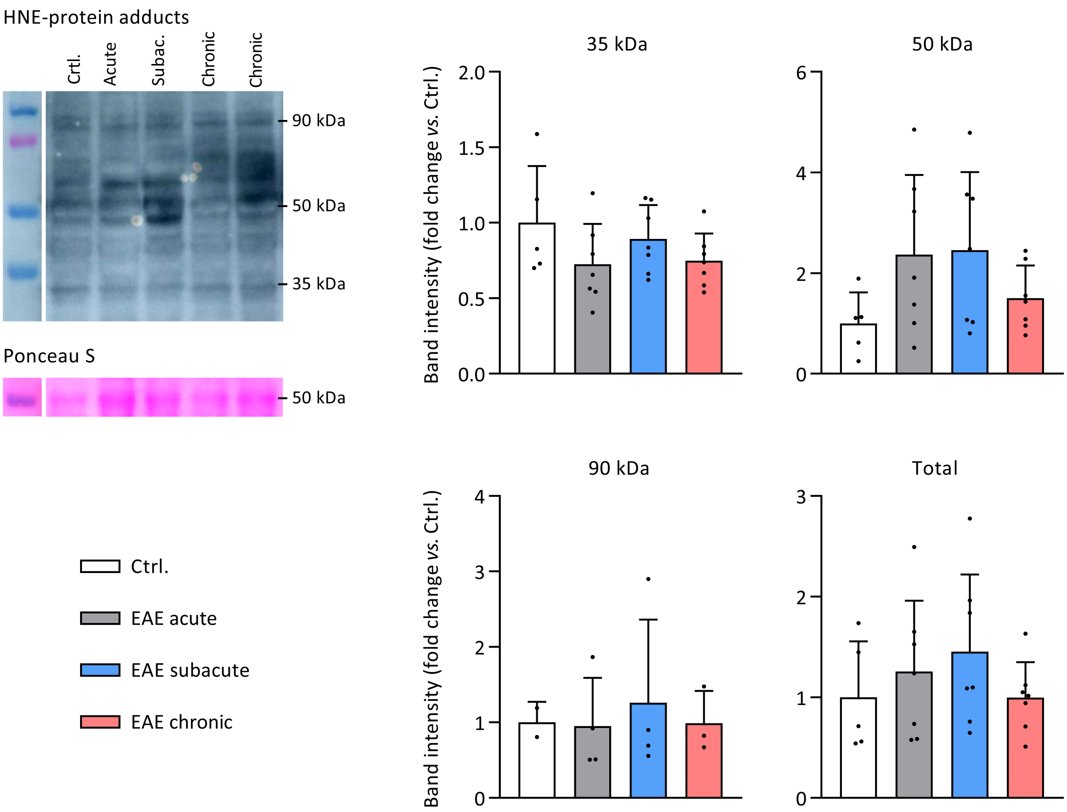
HNE-protein adducts
Crtl.
Acute
Subac.
Chronic
Chronic
90 kDa
50 kDa
35 kDa
Ponceau S
50 kDa
35 kDa
50 kDa
90 kDa
Total
Band intensity (fold change vs. Ctrl.)
Ctrl.
EAE acute
EAE subacute
EAE chronic

Figure S3

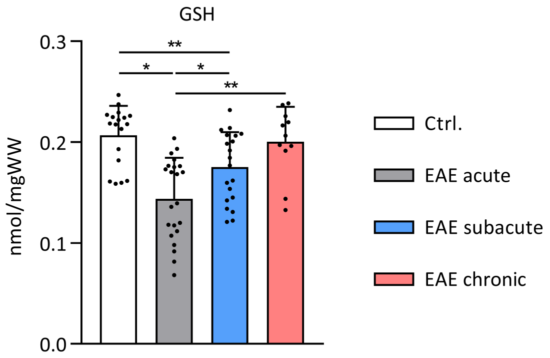
GSH
0.3
0.2
0.1
0.0
nmol/mgWW
**
*
*
**
Ctrl.
EAE acute
EAE subacute
EAE chronic

Figure S4

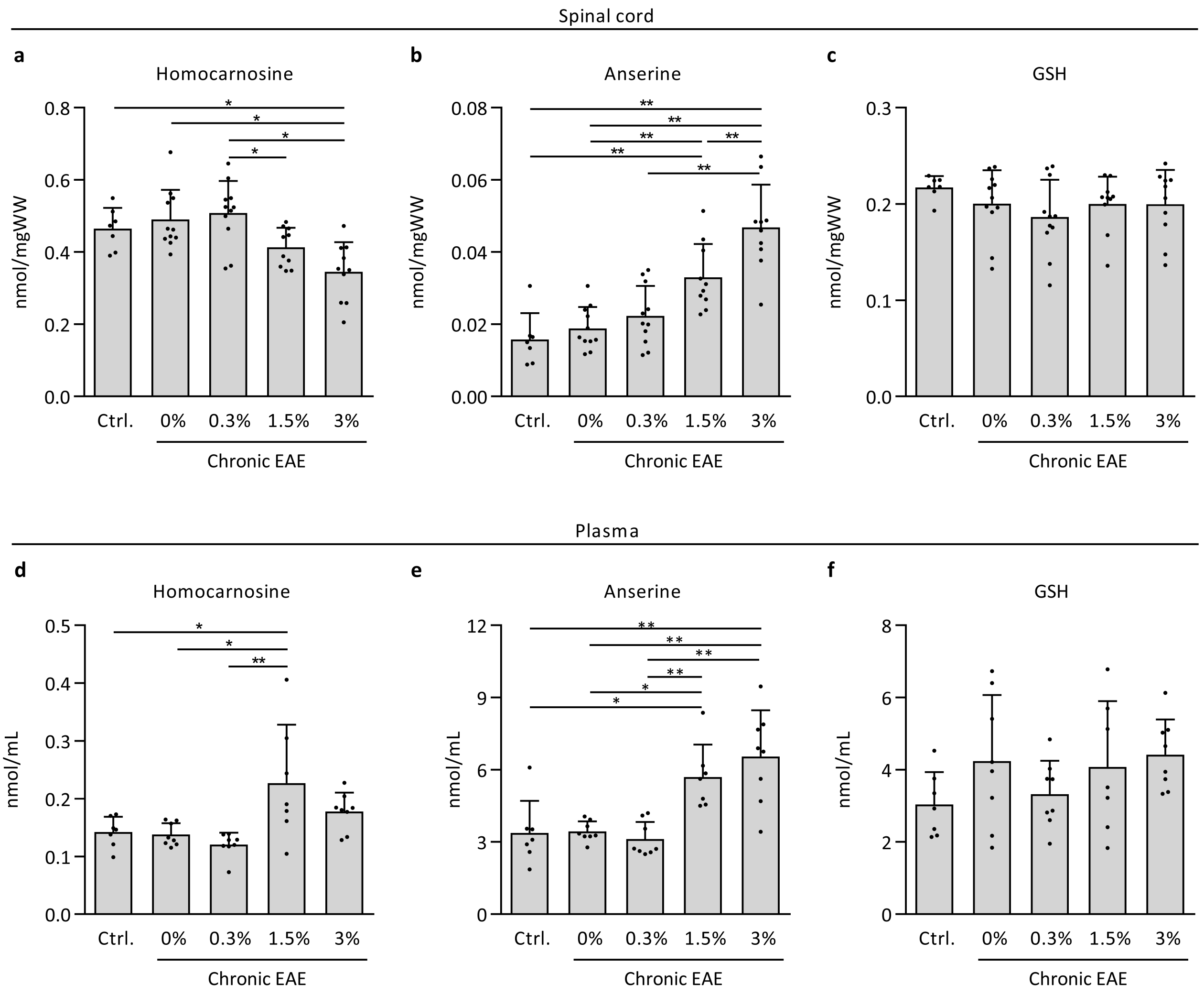

Spinal cord
a
Homocarnosine
nmol/mgWW
b
Anserine
nmol/mgWW
c
GSH
nmol/mgWW
Ctrl.
0%
0.3%
1.5%
3%
Chronic EAE
Plasma
d
Homocarnosine
nmol/mL
e
Anserine
nmol/mL
f
GSH
nmol/mL

Figure S5

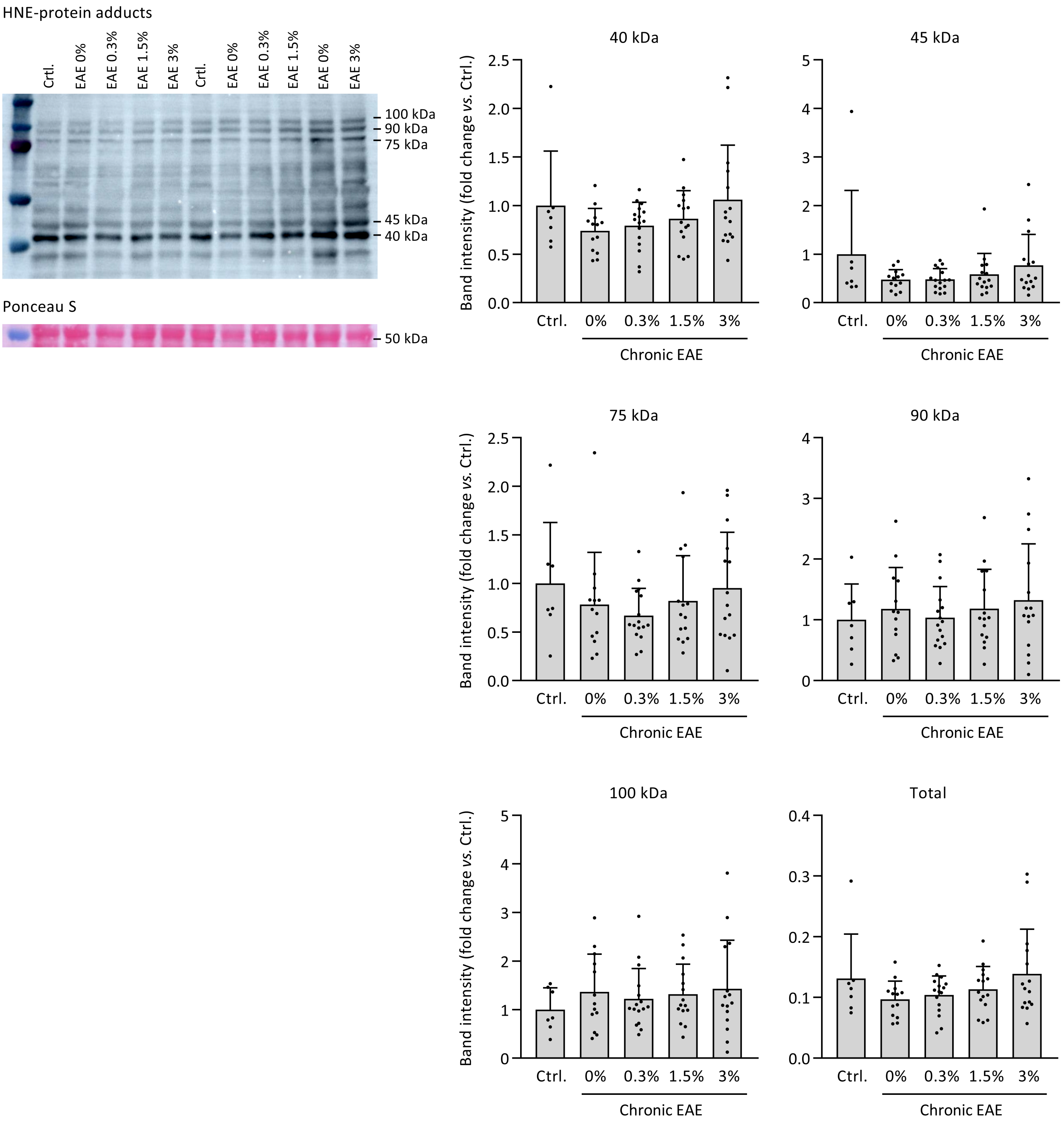
HNE-protein adducts
Crtl.
EAE 0%
EAE 0.3%
EAE 1.5%
EAE 3%
Crtl.
EAE 0%
EAE 0.3%
EAE 1.5%
EAE 0%
EAE 3%
100 kDa
90 kDa
75 kDa
45 kDa
40 kDa
Ponceau S
50 kDa
40 kDa
45 kDa
75 kDa
90 kDa
100 kDa
Total
Band intensity (fold change vs. Ctrl.)
Ctrl.
0%
0.3%
1.5%
3%
Chronic EAE

Figure S6

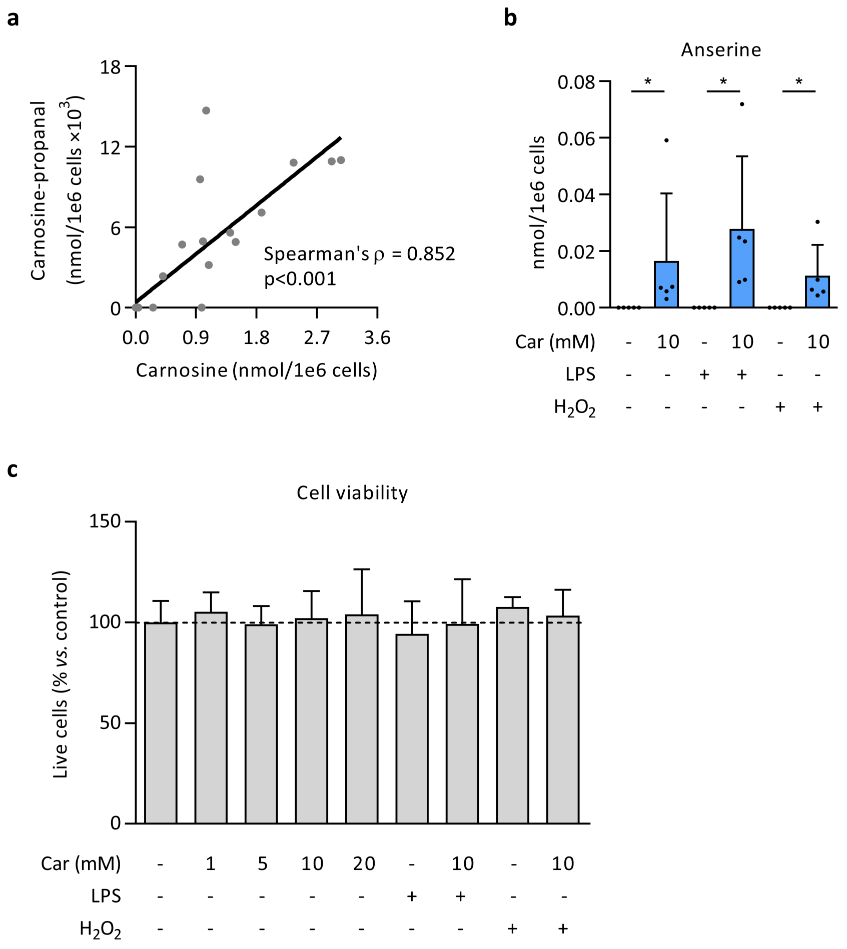
a
b
Anserine
0.08
0.06
0.04
0.02
0.00
nmol/1e6 cells
*
Car (mM) - 10 - 10 - 10
LPS - - + + - -
H2O2 - - - - + +
18
12
6
0
0.0
0.9
1.8
2.7
3.6
Carnosine-propanal (nmol/1e6 cells ×10^3)
Carnosine (nmol/1e6 cells)
Spearman's ρ = 0.852
p<0.001
c
Cell viability
150
100
50
0
Live cells (% vs. control)
Car (mM) - 1 5 10 20 - 10 - 10
LPS - - - - - + + - -
H2O2 - - - - - - - + +

Figure S7
